# A small-molecule inhibitor of BCL10-MALT1 interaction abrogates progression of diffuse large B cell lymphoma

**DOI:** 10.1172/JCI164573

**Published:** 2025-04-15

**Authors:** Heejae Kang, Lisa M. Maurer, Jing Cheng, Mei Smyers, Linda R. Klei, Dong Hu, Juliana Hofstatter Azambuja, Marcelo J. Murai, Ahmed Mady, Ejaz Ahmad, Matthew Trotta, Hanna B. Klei, Minda Liu, Prasanna Ekambaram, Zaneta Nikolovska-Coleska, Bill B. Chen, Linda M. McAllister-Lucas, Peter C. Lucas

**Affiliations:** 1 Department of Pathology and; 2 Department of Pediatrics, University of Pittsburgh School of Medicine, Pittsburgh, Pennsylvania, USA.; 3 Department of Laboratory Medicine and Pathology, Mayo Clinic, Rochester, Minnesota, USA.; 4 Department of Pathology, University of Michigan Medical School, Ann Arbor, Michigan, USA.; 5 Department of Medicine, University of Pittsburgh School of Medicine, Pittsburgh, Pennsylvania, USA.; 6 UPMC Hillman Cancer Center, Pittsburgh, Pennsylvania, USA.; 7 Department of Pediatrics and Adolescent Medicine, Mayo Clinic, Rochester, Minnesota, USA.; 8 Mayo Clinic Comprehensive Cancer Center, Rochester, Minnesota, USA.

**Keywords:** Immunology, Oncology, Therapeutics, Lymphomas, NF-kappaB, Signal transduction

## Abstract

Diffuse large B cell lymphoma (DLBCL) is the most common type of non-Hodgkin lymphoma, and the activated B cell–like subtype (ABC-DLBCL) is associated with particularly poor outcome. Many ABC-DLBCLs harbor gain-of-function mutations that cause inappropriate assembly of the CARMA1-BCL10-MALT1 (CBM) signalosome, a cytoplasmic complex that drives downstream NF-κB signaling. MALT1 is the effector protein of the CBM signalosome such that its recruitment to the signalosome via interaction with BCL10 allows it to exert both protease and scaffolding activities that together synergize in driving NF-κB. Here, we demonstrate that a molecular groove located between two adjacent immunoglobulin-like domains within MALT1 represents a binding pocket for BCL10. Leveraging this discovery, we performed an in silico screen to identify small molecules that dock within this MALT1 groove and act as BCL10-MALT1 protein-protein interaction (PPI) inhibitors. We report the identification of M1i-124 as a first-in-class compound that blocks BCL10-MALT1 interaction, abrogates MALT1 scaffolding and protease activities, promotes degradation of BCL10 and MALT1 proteins, and specifically targets ABC-DLBCLs characterized by dysregulated MALT1. Our findings demonstrate that small-molecule inhibitors of BCL10-MALT1 interaction can function as potent agents to block MALT1 signaling in selected lymphomas, and provide a road map for clinical development of a new class of precision-medicine therapeutics.

## Abstract 2

This study describes the discovery and characterization of a novel inhibitor of the CARMA1-BCL10-MALT1 signaling pathway and its potential use in the treatment of lymphoma.

## Graphical abstract

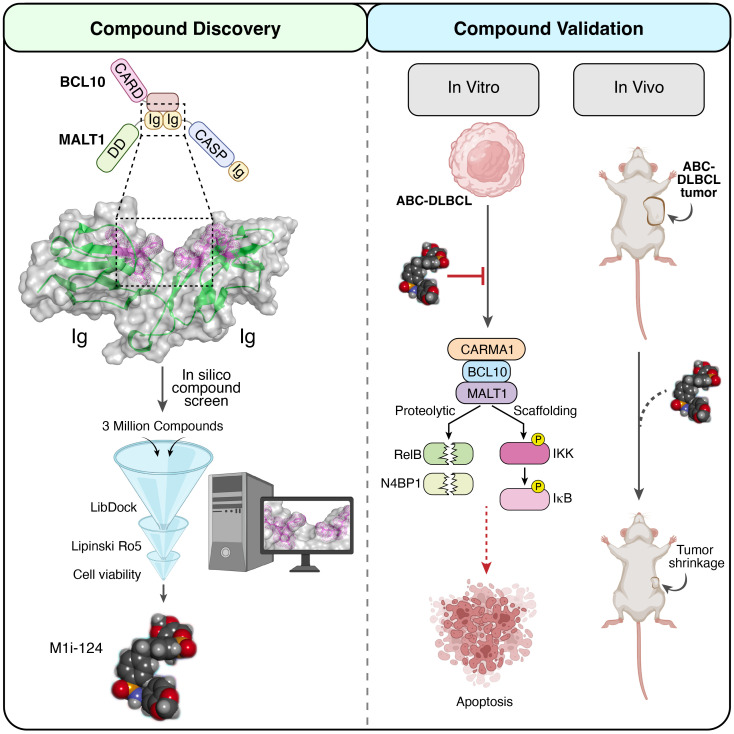

## Introduction

Diffuse large B cell lymphoma (DLBCL) is the most common type of non-Hodgkin lymphoma, accounting for approximately 30% of newly diagnosed cases (1). A conventional classification scheme categorizes DLBCL into the germinal center B cell–like (GCB) and activated B cell–like (ABC) subtypes based on cell of origin (1, 2). Compared with GCB-DLBCL, ABC-DLBCL remains difficult to treat, with 5-year progression-free survival of around 40% even after aggressive immunochemotherapy with R-CHOP (rituximab, cyclophosphamide, doxorubicin, vincristine, prednisone) (1). Recently, the advent of molecular profiling has further refined the classification of DLBCL into subsets based on shared molecular features that better account for the high degree of genetic and phenotypic heterogeneity (3–6). An understanding of these subsets provides the opportunity to develop molecularly informed targeted therapies to supplement chemotherapy.

One subset of ABC-DLBCL is characterized by oncogenic mutations in proteins including the B cell receptor (CD79A/B) and CARMA1 that lead to inappropriate NF-κB transcription factor activation (7, 8). These oncogenic mutations drive dysregulated assembly of the CARMA1-BCL10-MALT1 (CBM) signalosome, wherein MALT1 functions as the effector component to activate downstream NF-κB signaling (7). In normal lymphocytes, antigen stimulation of the B or T cell receptor triggers the phosphorylation of CARMA1 by protein kinase C, and this leads to the assembly of the CBM signalosome (9, 10). Phosphorylated CARMA1 engages with BCL10 to nucleate the formation of a macromolecular BCL10 filament (11). MALT1, through direct interaction with BCL10, decorates the periphery of the BCL10 filament; local dimerization of MALT1 in the context of the growing filament then enables its activation and subsequent induction of the NF-κB signaling pathway (12, 13). In contrast to the regulated CBM assembly that occurs in antigen-stimulated lymphocytes, ABC-DLBCL cells harboring oncogenic mutations in CD79A/B or CARMA1 show constitutive assembly of the CBM signalosome and unrestrained NF-κB activity, which provides a potent proliferative and survival signal (4, 7).

**Figure 1:**
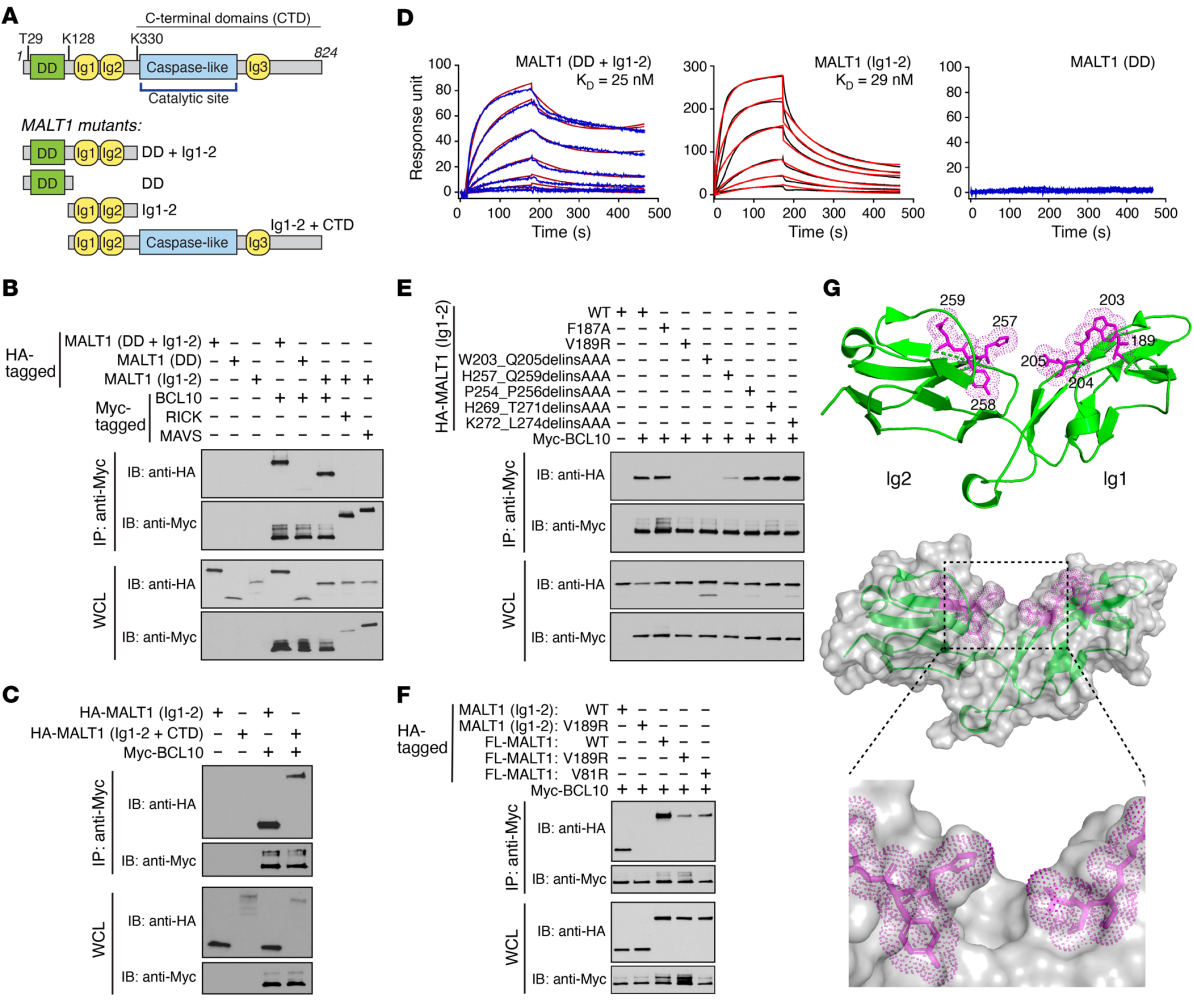
Identification of a BCL10-binding site at the junction of MALT1 Ig1 and Ig2 domains. (**A**) Schematic of full-length MALT1 protein highlighting the death domain (DD), immunoglobulin-like domains (Ig), and caspase-like proteolytic domain. Specific residues used to create deletion mutants are indicated. (**B** and **C**) Co-IP of HA-tagged MALT1 protein fragments with Myc-tagged BCL10. Myc-tagged RICK and MAVS served as negative controls. (**D**) SPR curves quantifying the binding of MALT1 protein fragments to immobilized BCL10, with calculated *K_D_* values. (**E**) Co-IP of HA-tagged MALT1(Ig1-2) harboring indicated mutations with Myc-tagged BCL10. (**F**) Co-IP of either HA-tagged full-length (FL) MALT1 or HA-tagged MALT1(Ig1-2) harboring indicated mutations with Myc-tagged BCL10. (**G**) The location of each mutation that disrupts binding of MALT1(Ig1-2) to BCL10 in co-IP experiments is highlighted in purple in the published MALT1(Ig1-2) crystal structure (PDB ID: 3K0W). Data in **B**–**F** are representative of a minimum of 3 independent repeats. All co-IP experiments were performed by expression of proteins in transiently transfected 293T cells.

MALT1 is a multidomain protein composed of a death domain (DD), 2 tandem immunoglobulin-like (Ig-like) domains, a caspase-like proteolytic domain, and a third Ig-like domain near the C-terminus (Figure 1A) (12). As the effector protein of the CBM signalosome, MALT1 has two main functions, acting as a scaffolding molecule and as a protease. As a scaffold, MALT1 recruits downstream signaling proteins, including TNF receptor–associated factor 6 (TRAF6), which promote the direct activation of the inhibitor of κB (IκB) kinase complex (IKK complex), a master regulator of NF-κB (14, 15). As a protease, MALT1 cleaves and destroys multiple substrates via a caspase-like enzymatic domain that contains an essential catalytic cysteine (16). Several of these substrates, including RelB and N4BP1, are negative regulators of NF-κB so that MALT1 protease activity serves to augment the NF-κB signal that is induced directly through its scaffolding activity (17–19). The convergence of these two independent mechanisms of action therefore allows MALT1 to drive a potent and sustained NF-κB survival signal (7, 20).

Recognition that MALT1 is a central driver of ABC-DLBCL lymphomagenesis has fueled the development of multiple small-molecule inhibitors of MALT1 proteolytic activity, including inhibitors that bind to the active site within the caspase-like proteolytic domain, as well as allosteric MALT1 protease inhibitors (21). These MALT1 protease inhibitors have been shown to effectively suppress tumor growth in xenograft models of ABC-DLBCL (22–24). Early-phase clinical trials investigating several MALT1 protease inhibitors in relapsed or refractory B cell non-Hodgkin lymphoma (including DLBCL) and chronic lymphocytic leukemia are under way (ClinicalTrials.gov NCT05618028, NCT03900598, NCT04876092, NCT04657224, NCT05515406, and NCT05544019). Nevertheless, clinical use of MALT1 protease inhibitors could be limited by (a) incomplete blockade of MALT1-dependent NF-κB activity within tumor cells due to the inability of protease inhibitors to abrogate MALT1 scaffolding activity, and (b) unintended systemic autoimmunity and multiorgan inflammation that can be seen in the setting of selective MALT1 protease inhibition without concomitant inhibition of MALT1 scaffolding activity (25–29). This inflammation is thought to be due to an imbalance of regulatory T and effector T cells that occurs with sustained MALT1 protease inhibition, wherein Tregs are preferentially impaired and effector memory T cells are increased, as are levels of the proinflammatory cytokine IFN-γ (25–31). These factors prompted us to launch a drug discovery campaign to identify candidate small molecules that might disrupt both MALT1 scaffolding and protease activities. We decided to focus our efforts on identifying molecules that could act to block BCL10-MALT1 interaction, which is fundamental to productive CBM complex assembly and critical to enabling both aspects of MALT1 function.

The interaction between MALT1 and BCL10 has been intensively investigated for years, and there is increasing appreciation for its complexity and for the notion that the nature of the interaction may change depending on the state of cell activation. In resting lymphocytes, MALT1 is constitutively bound to BCL10, likely forming heterodimers or relatively low–molecular weight complexes (11, 13, 32). Previous work implicated the MALT1 tandem Ig-like domains (Ig1-2) as playing a crucial role in the BCL10-MALT1 interaction under these conditions (33–35). In contrast, cryogenic electron microscopy studies of CBM complex activation and macromolecular filament formation clearly demonstrated that MALT1 decorates BCL10 filaments through a direct interaction between the MALT1 DD and the BCL10 caspase recruitment domain (CARD), with the MALT1 Ig1-2 domains disengaged from direct interaction with the core BCL10 filament (13). Together, these observations suggest that BCL10 and MALT1 proteins initially interact via the MALT1 Ig1-2 domains and then undergo an interaction transition once BCL10-MALT dimers are recruited into CBM filaments.

Here, we identify a specific molecular groove located at the junction of the MALT1 Ig1 and Ig2 domains that represents a relatively small, and potentially druggable, BCL10 interaction interface. Using an in silico compound screen targeting this interaction site, we identified what we believe to be a first-in-class BCL10-MALT1 protein-protein interaction (PPI) inhibitor, M1i-124. Consistent with its ability to inhibit the BCL10-MALT1 interaction, this compound blocks both MALT1 protease and scaffolding activities and also potently drives a decrease in BCL10 and MALT1 protein levels within ABC-DLBCL cells, likely due to disruption of the self-stabilizing interaction of the BCL10 and MALT1 proteins (36, 37). M1i-124 selectively inhibits NF-κB signaling, cytokine production, and proliferation of MALT1-dependent ABC-DLBCL cells without affecting MALT1-independent GCB-DLBCL cells, promotes selective ABC-DLBCL cell death, and inhibits ABC-DLBCL tumor growth in xenograft models. This study provides important insight into the nature of the interaction between BCL10 and MALT1 and advances a strategy for simultaneously targeting MALT1 protease and scaffolding functions for the treatment of MALT1-dependent lymphoid malignancies.

## Results

### Identification of the BCL10-binding site within MALT1(Ig1-2).

To pursue the goal of identifying a BCL10-MALT1 PPI inhibitor, we first sought to determine whether an interaction interface exists within either BCL10 or MALT1 that might be amenable to small-molecule PPI targeting. Using coimmunoprecipitation (co-IP), we demonstrated that a MALT1 fragment containing the DD and Ig1-2 domains (DD+Ig1-2; Figure 1A) bound to BCL10 (Figure 1B), consistent with previously published results (33). Importantly, we found that a MALT1 fragment containing only the Ig1-2 domains also bound efficiently to BCL10, whereas a MALT1 fragment containing only the DD demonstrated minimally detectable binding (Figure 1B). BCL10 is a member of a family of CARD-containing proteins (12), so to confirm that MALT1(Ig1-2) binding is specific for BCL10, we tested other family members in the co-IP assay and found that MALT1(Ig1-2) did not bind to the CARD-containing proteins RICK and MAVS (Figure 1B). A fragment of MALT1 consisting of the Ig1-2 domains and the remaining C-terminus (Ig1-2+CTD), which lacks only the DD, also bound strongly to BCL10 (Figure 1C). These results support the notion that when BCL10 and MALT1 proteins are expressed in unstimulated cells in the absence of coexpressed CARMA1, the major interface for BCL10-MALT1 interaction occurs through the tandem MALT1 Ig-like domains rather than through the DD, in contrast to what is observed when BCL10 is assembled into filaments.

Next, we sought to determine the binding affinity of MALT1(DD+Ig1-2), MALT1(Ig1-2), and MALT1(DD) for BCL10 using surface plasmon resonance (SPR) (Supplemental Figure 1, A and B; supplemental material available online with this article; https://doi.org/10.1172/JCI164573DS1). We found that MALT1(DD+Ig1-2) and MALT1(Ig1-2) bound to immobilized BCL10 with high affinity (*K_D_* of 25 nM and 29 nM, respectively), whereas MALT1(DD) exhibited no detectable binding to BCL10 (Figure 1D). In the SPR assay, BCL10 was immobilized on the sensor chip in a manner that likely prevented filament formation and retained BCL10 in the monomeric form, so that these results further support the conclusion that the MALT1 Ig1-2 domains have dramatically higher affinity for monomeric BCL10 than does the MALT1 DD.

To further evaluate the ability of MALT1(Ig1-2) to bind to monomeric BCL10, we tested for BCL10-MALT1 interaction in BCL10^–/–^ cells reconstituted with the E53R mutant of BCL10. Previous reports indicate that the BCL10 E53R mutation, which resides within the BCL10 CARD, prevents BCL10 self-oligomerization and maintains BCL10 entirely in its monomeric form (11, 32). We confirmed this observation in our own co-IP system by showing that the BCL10 E53R mutant was completely incapable of binding to itself, unlike WT BCL10, which strongly self-associated (Supplemental Figure 1C). In contrast, we found that the BCL10 E53R mutant did bind to MALT1(Ig1-2) (Supplemental Figure 1D). Taken together, the multiple co-IP and SPR studies strongly argue that the MALT1 Ig1-2 domains contain a major interface that allows MALT1 to bind BCL10 in its monomeric, non-filamentous state.

To identify the precise site within MALT1(Ig1-2) that interacts with BCL10, we introduced single point mutations or blocks of 3 alanine residue substitutions into the MALT1(Ig1-2) fragment and evaluated the impact on BCL10 binding. We found that introducing mutations at the junction between Ig1 and Ig2 decreased binding to BCL10, whereas introducing mutations outside of this region did not alter binding activity. Specifically, we observed a marked decrease in BCL10 binding caused by a V189R point mutation as well as by alanine substitutions spanning amino acids 203–205 or 257–259 (Figure 1E and Supplemental Figure 1E). To verify the importance of this region, we introduced the V189R mutation into the full-length MALT1 protein and again observed that the V189R mutation caused substantial loss of BCL10 binding (Figure 1F). Interestingly, the effect of the V189R mutation was somewhat less dramatic in the context of the full-length MALT1 protein as compared with the context of the isolated MALT1 Ig1-2 fragment. One possible explanation for this difference is that the BCL10 interaction observed in co-IPs using full-length MALT1 reflects a mix of interactions between MALT1 and both the monomeric and oligomeric forms of BCL10. Thus, the residual BCL10 interaction observed with full-length MALT V189R may represent DD-dependent interactions of MALT1 with a proportion of the expressed BCL10 that has undergone oligomerization in the cell. Supporting this concept, we found that full-length MALT harboring a V81R substitution in the DD, which has previously been shown to disrupt the interaction between MALT1 and oligomerized BCL10 (13), also demonstrated reduced binding to BCL10 in the co-IP assay (Figure 1F). In summary, we find that amino acid residues clustered at the junction of Ig1 and Ig2 are important for association with BCL10. Surface representations of the crystal structure of the tandem Ig domains (Protein Data Bank [PDB] ID: 3K0W) reveal that these amino acids surround a deep hydrophobic groove in the protein that could represent a druggable binding pocket for BCL10 (Figure 1G).

### In silico screening to identify small molecules that disrupt BCL10-MALT1 interaction.

**Figure 2:**
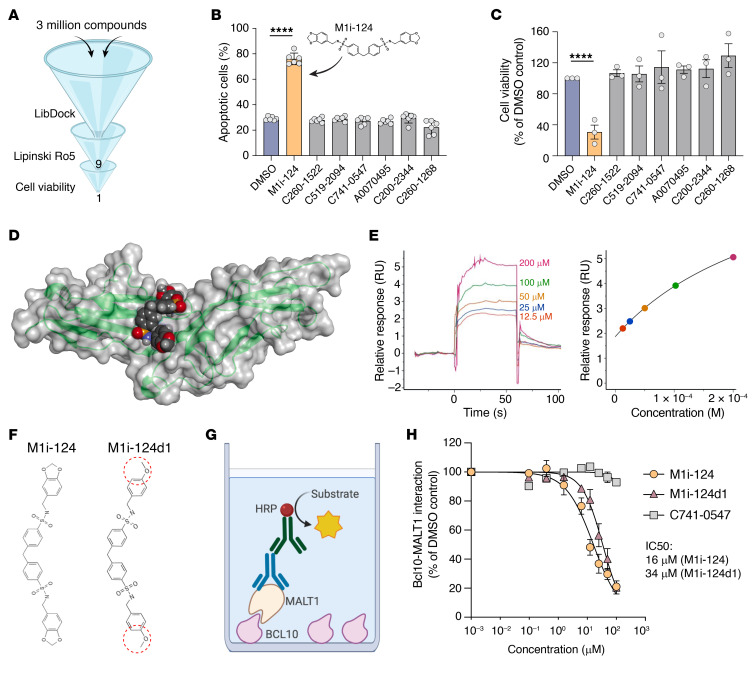
In silico drug screen identifies M1i-124 as a BCL10-MALT1 PPI inhibitor. (**A**) Schematic of in silico drug screen. Three million compounds were screened using LibDock followed by application of Lipinski’s rule of 5 (Ro5) filters, which led to the identification of 9 small-molecule candidates, 7 of which were commercially available. (**B**) Apoptosis of TMD8 cells, quantified by flow cytometric analysis of annexin V/SYTOX Blue staining, after 6 days of treatment with 1 μM of the 7 candidate molecules from the LibDock screen (mean ± SEM; *n* = 6). Structure of the only active compound, M1i-124, is shown. (**C**) Viability of TMD8 cells after 8 days of treatment with 1 μM of the 7 commercially available molecules (mean ± SEM; *n* = 3). (**D**) Lead compound, M1i-124, is docked onto the groove between Ig1 and Ig2 near the proposed BCL10-binding site with MALT1(Ig1-2) (PDB ID: 3K0W). (**E**) SPR analysis of the binding of M1i-124 to immobilized full-length MALT1 confirmed target engagement using a steady-state affinity model. (**F**) Structures of lead compound, M1i-124, and its analog, M1i-124d1. (**G** and **H**) ELISA schematic (**G**) and analysis (**H**) showing that M1i-124 and M1i-124d1, but not C741-0547, inhibit the binding of MALT1 to immobilized BCL10 in a dose-dependent manner with indicated IC_50_ values (mean ± SEM; *n* = 2–3). For **B** and **C**, statistical analyses were performed using a 1-way ANOVA with Dunnett’s multiple-comparison test. ****P < 0.0001.

We next focused on identifying small molecules that might dock within the BCL10-binding groove of the tandem MALT1 Ig-like domains, with the long-term goal of developing a clinically viable BCL10-MALT1 PPI inhibitor. Using this region as a virtual docking site, we performed an in silico screen of 3 million compounds from the ChemDiv chemical libraries, using the LibDock module of Discovery Studio 3.5 (38) to identify those molecules with potential affinity for the docking site based on geometric, energy, and chemical environment complementarity. To enrich for compounds with drug-like properties, we applied Lipinski’s rule of 5 (Ro5) filters (39), which ultimately led to the identification of 9 candidate compounds, 7 of which were commercially available (Figure 2A). We then tested the ability of the 7 compounds to induce apoptosis and reduce the overall viability of a MALT1-dependent ABC-DLBCL cell line (TMD8), using flow cytometric analysis of annexin V staining and CellTiter-Glo ATP assays, respectively. Of the 7 compounds tested, only M1i-124 induced significant apoptosis and loss of cell viability (Figure 2, B and C).

Next, we evaluated MALT1 target engagement by M1i-124. In silico modeling suggests that M1i-124 docks within a hydrophobic pocket located between the MALT1 Ig1 and Ig2 domains (Figure 2D), via several putative electrostatic and van der Waals interactions (Supplemental Figure 2). SPR analysis of the binding affinity of M1i-124 to immobilized full-length MALT1 yielded an equilibrium dissociation constant (*K_D_*) of 2.96 × 10^–4^ M, experimentally confirming target engagement (Figure 2E). Through a limited structure-activity relationship study, we identified one analog of interest, M1i-124d1, which differs from M1i-124 in that the 1,3-dioxolane groups at the terminal ends of the molecule are replaced with 4-methoxy groups (Figure 2F). We then established an ELISA-based readout to directly test the ability of these compounds to inhibit the interaction between MALT1 and BCL10 (Figure 2G). The specificity of this assay was confirmed by showing that while full-length MALT1 bound to immobilized BCL10, another protein involved in CBM signaling (G protein–coupled receptor kinase 2 [GRK2]; ref. 40) did not bind to the immobilized BCL10 (Supplemental Figure 3A). Next, we preincubated full-length recombinant MALT1 for 1 hour with increasing concentrations of M1i-124 or M1i-124d1 and measured their effect on the ability of MALT1 to bind immobilized BCL10. Both M1i-124 and M1i-124d1 inhibited the interaction of MALT1 with BCL10 in a dose-dependent manner with IC_50_ of 16 μM and 34 μM, respectively (Figure 2H). Compound C741-0547, which had no effect on TMD8 cells in our initial apoptosis and viability screens (Figure 2, B and C), did not inhibit the interaction between MALT1 and BCL10 (Figure 2H). As an additional control, we tested the effects of M1i-124 and M1i-124d1 on the interaction between MyoD and inhibitor of DNA binding 1 (Id1) proteins using this ELISA-based platform and found that neither M1i-124 nor M1i-124d1 could inhibit the binding of Id1 to immobilized MyoD (Supplemental Figure 3B). These results establish M1i-124 and its analog M1i-124d1 as what we believe to be first-in-class BCL10-MALT1 PPI inhibitors, and lead compounds in our efforts to develop a new class of precision therapeutics to block dysregulated MALT1 signaling.

### M1i-124 and M1i-124d1 inhibit both proteolytic and scaffolding activities of MALT1 in stimulated T cells.

**Figure 3:**
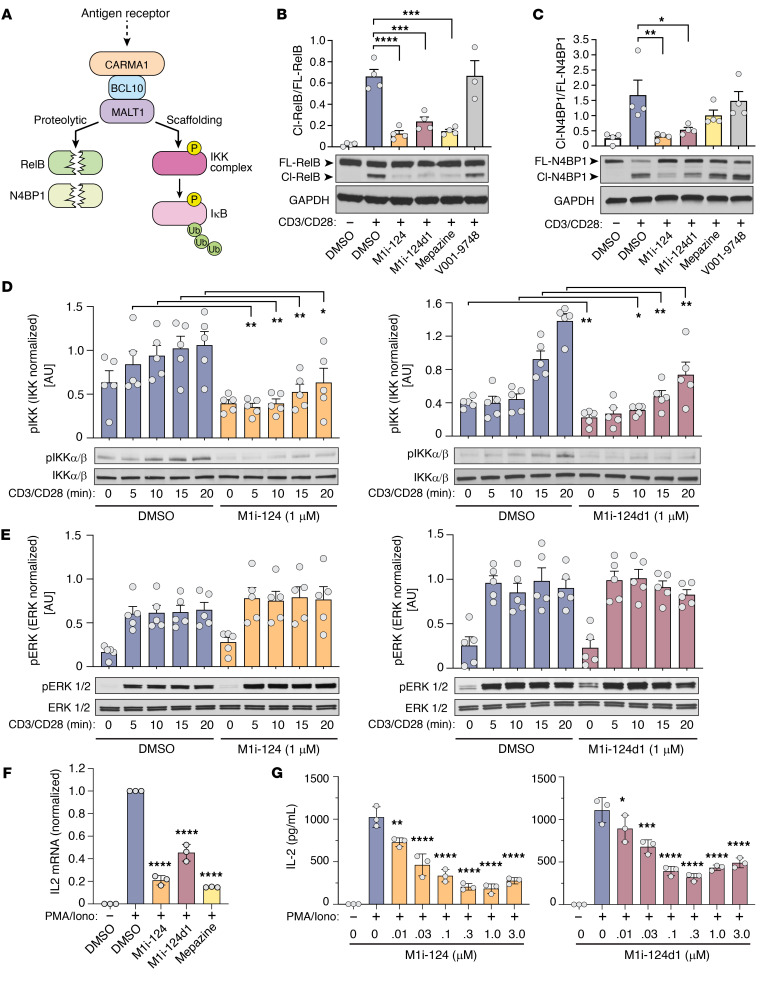
M1i-124 and M1i-124d1 inhibit MALT1 protease and scaffolding functions in stimulated Jurkat T cells. (**A**) Schematic of the CBM complex highlighting the 2 functions of MALT1. MALT1 proteolytic activity cleaves RelB and N4BP1, both of which are analyzed in this study. MALT1 scaffolding activity leads to activation of the IKK complex and phosphorylation of IκB. (**B** and **C**) Effect of 1 μM compound pretreatment on CD3/CD28-induced RelB and N4BP1 cleavage in Jurkat T cells. Representative Western blots showing full-length (FL) and cleaved (Cl) proteins are shown. Quantification of the Cl/FL ratio for multiple experiments is plotted. Statistical analyses were performed using 1-way ANOVA and Dunnett’s multiple-comparison test (mean ± SEM; *n* = 4). (**D** and **E**) Effect of 1 μM M1i-124 (**D**) and 1 μM M1i-124d1 (**E**) on IKKα/β and ERK phosphorylation following CD3/CD28 stimulation of Jurkat T cells. Representative Western blots are shown along with quantification of the phosphorylated-to-total protein ratios for multiple experiments. Unpaired *t* test was used to compare each stimulation time point (mean ± SEM; *n* = 5). (**F**) *IL2* mRNA induction in Jurkat T cells pretreated with 1 μM M1i-124, 1 μM M1i-124d1, or 5 μM mepazine and stimulated with PMA/Iono (mean ± SEM; *n* = 3). (**G**) Secreted IL-2 protein measured by ELISA in Jurkat T cells pretreated with M1i-124 or M1i-124d1 and stimulated with PMA/Iono (mean ± SEM; *n* = 3). For **F** and **G**, statistical analyses were performed using 1-way ANOVA and Dunnett’s multiple-comparison test. For all panels, *P < 0.05, **P < 0.01, ***P < 0.001, ****P < 0.0001.

BCL10-MALT1 interaction is a prerequisite for CBM complex assembly, and mutations in either protein that impair their association have been shown to abrogate both MALT1 scaffolding and proteolytic activities (13). As a scaffold, MALT1 recruits the ubiquitin ligase TRAF6, leading to direct activation of the IKK complex and subsequent rapid but transient NF-κB activation. In parallel, the protease activity of MALT1 cleaves and inactivates negative regulators of NF-κB, including RelB and N4BP1 (17, 18), contributing to sustained and amplified NF-κB activation (Figure 3A) (7).

To evaluate the ability of M1i-124 and M1i-124d1 to inhibit MALT1 protease activity, we compared the cleavage of the MALT1 substrates RelB and N4BP1 with or without compound treatment in stimulated Jurkat T cells. We found that both M1i-124 and M1i-124d1 suppressed the cleavage of RelB and N4BP1 induced by anti-CD3/CD8 to an extent similar to that of mepazine, a known MALT1 protease inhibitor (Figure 3, B and C). A control compound, V001-9748, which was identified as a candidate in our in silico screen but failed to inhibit the BCL10-MALT1 interaction, did not inhibit RelB or N4BP1 cleavage (Figure 3, B and C). Next, we tested the ability of M1i-124 and M1i-124d1 to inhibit the scaffolding activity of MALT1 in anti-CD3/CD8–stimulated Jurkat T cells by measuring IKKα/β phosphorylation, and found that both compounds reduced IKK phosphorylation while ERK phosphorylation, known to be MALT1 independent (41), was unaffected (Figure 3, D and E).

IL-2 is an NF-κB–inducible cytokine secreted by T cells after T cell receptor ligation, and its production is known to be MALT1 dependent (42). To further confirm the inhibitory effects of M1i-124 or M1i-124d1 on MALT1 signaling, we analyzed the effect of compound treatment on the levels of *IL2* mRNA expression and IL-2 secretion in Jurkat T cells stimulated with phorbol 12-myristate 13-acetate (PMA)/ionomycin (Iono). Pretreatment with M1i-124 or M1i-124d1 for 4 hours prior to PMA/Iono stimulation led to a reduction in PMA/Iono–induced *IL2* gene expression (Figure 3F), as well as dose-dependent reduction in IL-2 secretion (Figure 3G). The degree of inhibition of IL-2 secretion by 1 μM M1i-124 was comparable to that by 5 μM mepazine (Supplemental Figure 4A). Similarly, 1 μM M1i-124 reduced IL-2 secretion from PMA/Iono– or anti-CD3/CD28–treated primary mouse splenocytes, again exhibiting a response comparable to the effect of 5 μM mepazine (Supplemental Figure 4B). Together, our results demonstrate that M1i-124 and M1i-124d1 inhibit both MALT1 protease and scaffolding activities, as well as MALT1-dependent cytokine expression and secretion in stimulated Jurkat T cells and primary splenocytes.

### M1i-124 and M1i-124d1 inhibit constitutive MALT1 activity in ABC-DLBCL cells.

**Figure 4:**
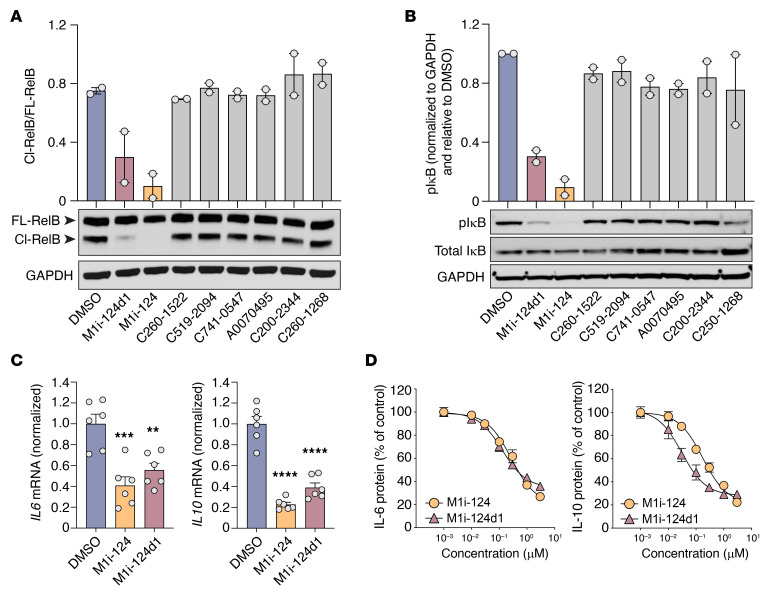
M1i-124 and M1i-124d1 inhibit MALT1 protease and scaffolding functions, and MALT1-dependent cytokine secretion, in ABC-DLBCL cells. (**A** and **B**) Effect of 1 μM compound pretreatment on constitutive RelB cleavage (**A**) and IκB phosphorylation (**B**) within TMD8 cells. Representative Western blots are shown along with quantification (mean ± SEM; *n* = 2). (**C**) *IL6* and *IL10* mRNA levels in TMD8 cells treated with 1 μM M1i-124 or M1i-124d1 (mean ± SEM; *n* = 6). (**D**) Dose-dependent inhibition of IL-6 and IL-10 secretion from TMD8 cells upon treatment with M1i-124 or M1i-124d1 (mean ± SEM; *n* = 3). Statistical analyses were performed using 1-way ANOVA and Dunnett’s multiple-comparison test. **P < 0.01, ***P < 0.001, ****P < 0.0001.

We next used the TMD8 cell line, an ABC-DLBCL line that harbors an oncogenic Y196H mutation in the B cell receptor CD79B subunit that confers dysregulated CBM-mediated NF-κB signaling (43), to test the ability of M1i-124 and M1i-124d1 to inhibit MALT1 protease and scaffolding functions in the context of constitutive MALT1 activation. As in stimulated Jurkat T cells, we found that treatment with M1i-124 or M1i-124d1 led to markedly reduced cleavage of RelB in TMD8 cells, while the other 6 compounds identified by our in silico screen had no effect (Figure 4A). In TMD8 cells, the degree of inhibition of RelB cleavage by 1 μM M1i-124 was similar to that seen with 5 μM mepazine (Supplemental Figure 5A). Treatment of TMD8 cells with either M1i-124 or M1i-124d1, but not with other candidate compounds from the in silico screen or with MALT1 protease inhibitor mepazine, also caused a marked reduction in IκB phosphorylation, suggesting inhibition of MALT1 scaffolding activity (Figure 4B and Supplemental Figure 5B). Thus, M1i-124 and M1i-124d are effective in inhibiting the constitutive MALT1 protease and scaffolding activities characteristic of TMD8 cells.

IL-6 and IL-10 are NF-κB–induced cytokines that promote the proliferation and survival of malignant B cells through both autocrine and paracrine mechanisms (44), and are negative prognostic factors in ABC-DLBCL (45, 46). ABC-DLBCL cell lines spontaneously secrete substantial levels of IL-6 and IL-10, whereas GCB-DLBCL cell lines express only a negligible amount of these cytokines (24). To evaluate the effect of M1i-124 and M1i-124d1 on NF-κB–dependent cytokine expression and secretion in ABC-DLBCL cells, we treated two ABC-DLBCL cell lines, TMD8 and OCI-Ly3, with 1 μM of M1i-124 or M1i-124d1 for 24 hours and found that this was sufficient to significantly decrease *IL6* and *IL10* mRNA levels (Figure 4C and Supplemental Figure 5C). Further, we observed a dose-dependent reduction of IL-6 and IL-10 secretion from both TMD8 and OCI-Ly3 cells when treated with either M1i-124 or M1i-124d1 (Figure 4D and Supplemental Figure 5D). The IC_50_ for inhibition of cytokine secretion by each compound in each cell line is reported in Supplemental Table 1.

### M1i-124 treatment leads to loss of BCL10 and MALT1 protein in ABC-DLBCL cells.

Surprisingly, the above studies revealed that the IC_50_ values for our most potent compound, M1i-124, were more than 10-fold lower in cell-based assays measuring functional MALT1 inhibition (e.g., IL-10/IL-6 secretion: 0.5–1.3 μM) as compared with cell-free assays of BCL10-MALT1 PPI inhibition (16 μM). This observation prompted us to investigate mechanisms that might explain why M1i-124 exhibits higher potency within cells as compared with cell-free assay systems. Previous studies demonstrated that depletion of cellular BCL10 protein leads to reduction of MALT1 protein levels, and vice versa (36, 37). This suggests that the cellular stability of each of the 2 binding partners depends on the presence of the other, such that BCL10-MALT1 heterodimers are more stable than uncomplexed, monomeric BCL10 or MALT1. Cellular MALT1 by itself has been shown to be unstable and subject to degradation via the ubiquitin-proteasome pathway (47). We therefore speculated that M1i-124, which inhibits BCL10-MALT1 interaction, might not only prevent the signaling events that depend on the BCL10-MALT1 interaction, but may also induce loss of both BCL10 and MALT1 proteins in the context of cell-based experiments. This effect on BCL10 and MALT1 protein stability would not occur in cell-free assay systems if cellular mechanisms for degradation, such as active proteasomes, are required.

**Figure 5:**
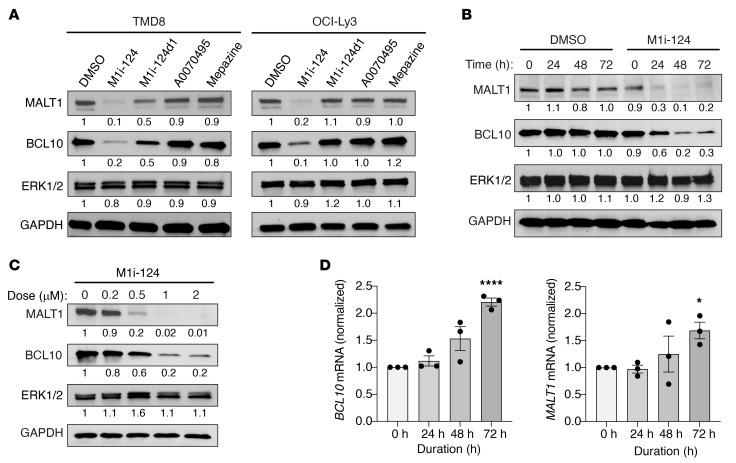
M1i-124 treatment leads to loss of BCL10 and MALT1 protein content in ABC-DLBCL cells. (**A**) Representative Western blots showing loss of BCL10 and MALT1 proteins, but not ERK1/2 protein, in TMD8 and OCI-Ly3 cells after 72 hours of treatment with 1 μM M1i-124. BCL10 or MALT1 loss was not observed after treatment with 1 μM of the negative control compound A0070495 or mepazine. (**B**) Representative Western blots showing time-dependent loss of BCL10 and MALT1 in TMD8 cells treated with 1 μM M1i-124. (**C**) Representative Western blots showing dose-dependent loss of BCL10 and MALT1 proteins in TMD8 cells treated with 1 μM M1i-124. For **A**–**C**, band quantifications, normalized for GAPDH, are indicated below each blot. (**D**) Time-dependent change in *BCL10* and *MALT1* mRNA expression levels with 1 μM M1i-124 treatment in TMD8 cells. Statistical analysis was performed using unpaired *t* test between 0 hours and 72 hours (mean ± SEM; *n* = 3). *P < 0.05, ****P < 0.0001.

To test the possibility that M1i-124 destabilizes BCL10 and MALT1 within the cell as a consequence of disrupting BCL10-MALT1 interaction, we treated ABC-DLBCL cell lines (TMD8, OCI-Ly3) with M1i-124 for 72 hours and measured the cellular levels of the BCL10 and MALT1 proteins by Western blotting. Strikingly, M1i-124 potently induced the loss of both MALT1 and BCL10 proteins in each cell line, driving almost complete loss of MALT1 and dramatically reducing BCL10 levels (Figure 5A). M1i-124d1 elicited a lesser effect in comparison with M1i-124, while neither the control compound A0070495 from our in silico screen nor the MALT1 protease inhibitor mepazine had any effect on the levels of MALT1 and BCL10 (Figure 5A). This finding is consistent with previous reports showing that MALT1 protein level is unaffected by treatment with MALT1 protease inhibitors (mepazine or “compound 3”) in DLBCL cells (22, 24). Importantly, we observed no change in ERK1/2 protein levels after treatment with M1i-124, suggesting that the ability of M1i-124 to induce protein loss is specific for BCL10 and MALT1 (Figure 5A). Further analyses revealed that M1i-124 induced BCL10 and MALT1 protein loss in a time- and dose-dependent manner (Figure 5, B and C). In contrast, we observed a modest time-dependent upregulation in both *BCL10* and *MALT1* mRNA levels in TMD8 cells treated with M1i-124, indicating that the observed protein loss is not due to transcriptional inhibition and that cells incur sustained protein loss despite what may represent feedback transcriptional upregulation (Figure 5D).

### M1i-124 and M1i-124d1 selectively inhibit ABC-DLBCL cell proliferation and survival.

**Figure 6:**
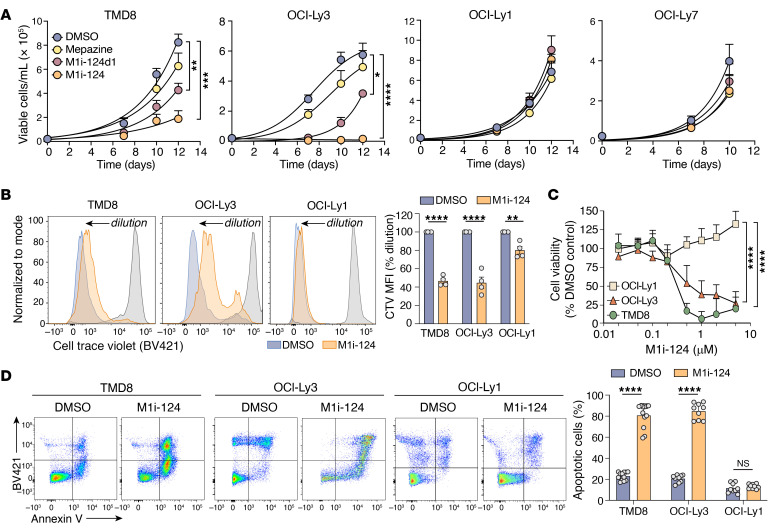
M1i-124 selectively inhibits the proliferation and survival of MALT1-dependent ABC-DLBCL cells. (**A**) Proliferation curves for ABC-DLBCL cell lines (TMD8 and OCI-Ly3) and GCB-DLBCL cell lines (OCI-Ly1 and OCI-Ly7) treated with 1 μM M1i-124, M1i-124d1, or mepazine. Statistical analyses were performed using 1-way ANOVA and Dunnett’s test at day 10–12 (mean ± SEM; *n* = 3–6). (**B**) CellTrace Violet dilution/cell division assay for TMD8, OCI-Ly3, and OCI-Ly1 cells treated with 1 μM M1i-124. Cell division profiles were quantified at day 6 and are plotted at right. Statistical analyses were performed using unpaired *t* tests (mean ± SEM; *n* = 4). (**C**) Viability of TMD8, OCI-Ly3, and OCI-Ly1 cells treated with increasing doses of M1i-124. TMD8 cells were slightly more sensitive to M1i-124 than were OCI-Ly3 cells (50% cytotoxic concentration ~0.2 μM vs. ~0.5 μM, respectively). Statistical analyses were performed using 2-way ANOVA with Tukey’s multiple-comparison test (mean ± SEM; *n* = 3-4). (**D**) Flow cytometric detection of apoptosis via annexin V/SYTOX Blue staining of DLBCL cells treated with M1i-124 for 8 days (TMD8 and OCI-Ly1 cells) and for 5 days (OCI-Ly3 cells). Representative flow panels are shown with quantification plotted at right (mean ± SEM; *n* = 9–12). For all panels, *P < 0.05, **P < 0.01, ***P < 0.001, ****P < 0.0001.

Next, we sought to determine whether M1i-124 and M1i-124d1 exhibit selective cytotoxicity against MALT1-dependent lymphoma cells. We first treated ABC-DLBCL (TMD8, OCI-Ly3) or GCB-DLBCL (OCI-Ly1, OCI-Ly7) cell cultures with a single 1 μM dose of M1i-124, M1i-124d1, mepazine, or DMSO, and then followed the proliferation of these lymphoma lines over the next 12 days. We found that treatment with either M1i-124 or M1i-124d1 led to significant reduction in the expansion of the MALT1-dependent ABC-DLBCL cultures while having no effect on expansion of the MALT1-independent GCB-DLBCL cultures (Figure 6A). M1i-124 was more effective than M1i-124d1 in this context, which might be explained by its greater potency in driving sustained loss of cellular MALT1 and BCL10 protein levels over time (see Figure 5A). Because of its greater efficacy in this assay, we focused on M1i-124 for subsequent analyses.

Because M1i-124 dramatically impairs expansion of ABC-DLBCL cultures, we speculated that the compound may be acting through a combination of mechanisms that could include the reduction of cell division and the promotion of cell death through induced apoptosis. To test for an effect on cell division, we treated ABC-DLBCL (TMD8, OCI-Ly3) and GCB-DLBCL (OCI-Ly1) cells with 1 μM M1i-124 and then monitored cell division over the ensuing 6 days by tracking the dilution of CellTrace Violet (Thermo Fisher Scientific) with flow cytometry. Using this approach, we found that treatment with M1i-124 substantially reduced TMD8 and OCI-Ly3 cell division, while having a minimal effect on OCI-Ly1 cell division (Figure 6B). We then directly measured the effect of M1i-124 on the viability of DLBCL cells by quantifying the level of ATP bioluminescence, and found that M1i-124 caused a dose-dependent decrease in viability of both ABC-DLBCL cell lines, but not OCI-Ly1 cells (Figure 6C). To determine whether the loss of viability was due to selectively induced apoptosis, we treated ABC-DLBCL (OCI-Ly3, TMD8) and GCB-DLBCL (OCI-Ly1) cells with 1 μM M1i-124 for up to 8 days and quantified apoptotic cells by annexin V and SYTOX Blue (Thermo Fisher Scientific) staining. Indeed, the results indicated that M1i-124 treatment selectively induced apoptosis of TMD8 and OCI-Ly3 cells, but not OCI-Ly1 cells (Figure 6D). Similarly to OCI-Ly1 cells, two other GCB-DLBCL lines (OCI-Ly7 and BJAB) showed either no or minimal apoptotic response to M1i-124 (Supplemental Figure 6A). As an additional assessment of specificity, we also tested the effect of M1i-124 on apoptosis of human primary T cells and saw no difference in comparison with untreated control (Supplemental Figure 6B). Together, these results demonstrate that M1i-124 inhibits both cell proliferation and viability of MALT1-dependent ABC-DLBCL cells, but not MALT1-independent GCB-DLBCL cells, and that the effect on ABC-DLBCL cells is at least partially due to induction of apoptosis.

### M1i-124 displays favorable drug-like properties.

**Figure 7:**
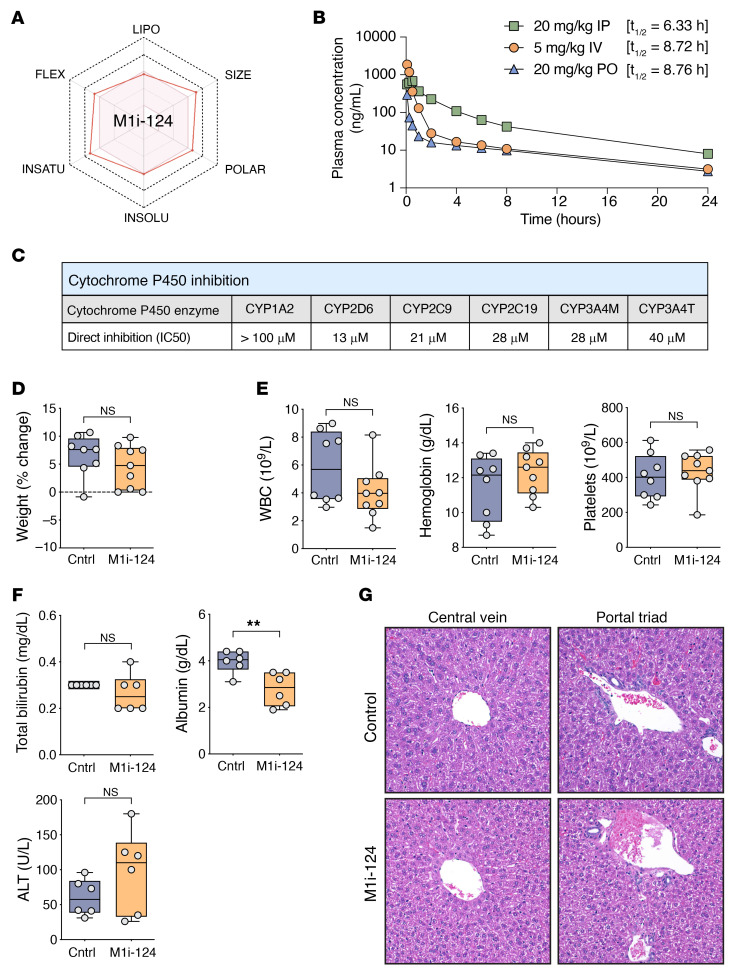
M1i-124 displays favorable drug-like properties and has no appreciable in vivo toxicity. (**A**) SwissADME modeling of M1i-124 shows minor deviations from the optimal range of physicochemical properties that predict bioavailability. (**B**) Plasma concentration of M1i-124 over 24 hours in mice treated with 5 mg/kg i.v., 20 mg/kg i.p., or 20 mg/kg p.o. M1i-124. (**C**) IC_50_ values for M1i-124–dependent inhibition of cytochrome P450 enzymes. (**D**–**G**) C57BL/6 mice were treated with either M1i-124 or vehicle control for 28 days. Body weight change from baseline is shown (**D**), along with results from complete blood count (**E**) and serum analyses (**F**). (**G**) Representative H&E images of liver from mice after 28 days of treatment. Original magnification, ×20. Statistical analyses were performed using unpaired *t* test (mean ± SEM; *n* = 6–9). **P < 0.01.

Pharmacokinetic and off-target profiling can provide valuable information for the potential therapeutic dosing and safety of an early-stage drug candidate and can guide next steps in preclinical drug optimization. For our candidate compound, M1i-124, we first evaluated the predicted bioavailability using SwissADME modeling (48). With this computational tool, 6 physicochemical properties of a compound — lipophilicity, size, polarity, solubility, flexibility, and saturation — are displayed on hexagonal axes, called the bioavailability radar. The pink shaded area within the hexagon represents the ideal range for each property that contributes to optimal oral bioavailability (Figure 7A). M1i-124 exhibits minor deviations from the optimal parameters for size, polarity, solubility, flexibility, and saturation. M1i-124 has a molecular weight (MW) of 594.66 g/mol, which is greater than the ideal MW of 500 g/mol or less. The topological polar surface area (TPSA) of M1i-124 is 146 Å^2^, greater than the ideal TPSA of 140 Å^2^ or less, which correlates with increased permeation rate (49). Using the estimated solubility (ESOL) model (50), the logS of M1i-124 is –6.07, which marginally exceeds the ideal logS of 6 or less for solubility. For assessment of molecular flexibility, M1i-124 has 10 rotatable bonds, which is 1 more than the ideal 9 or fewer rotatable bonds. The carbon bond saturation of M1i-124 is 0.17, which is lower than the optimal saturation of 0.25 or higher. The carbon bond saturation property has been shown to correlate with solubility, and also represents one surrogate measure of the complexity of a molecule, which is associated with target selectivity (51). For lipophilicity, the consensus logP_o/w_ (octanol-water partition coefficient) averaged from 5 different predictive models is 3.77 (i.e., 10:3.77 partitioning in organic/aqueous phases), within the preferred range, indicating that M1i-124 exhibits a balanced aqueous and lipid solubility. Overall, improvements that could be made in the physicochemical properties for optimal bioavailability, based on the SwissADME predictive modeling, include reductions in the size, polar surface area, and rotatable bonds, as well as enhanced solubility and saturation.

To further evaluate M1i-124, we performed in vivo pharmacokinetic (PK) studies in mice using i.v., i.p., and p.o. administration of the compound (Figure 7B). Following i.v. injection of 5 mg/kg M1i-124, the plasma concentration of the compound declined in a multiphasic manner from a mean initial concentration of 1,864 ng/mL with a terminal half-life (*t*_1/2_) of 8.72 hours. M1i-124 displayed systemic clearance of 77.9 mL/min/kg and a high steady-state volume of distribution (V_ss_) of 15.4 L/kg, suggesting extensive metabolism and tissue distribution. Following i.p. injection of 20 mg/kg M1i-124, the compound reached mean peak plasma concentration (C_max_) of 681 ng/mL within 30 minutes, then declined in a multiphasic manner with a *t*_1/2_ of 6.33 hours. Within 5 minutes of oral administration of 20 mg/kg M1i-124, the compound reached a C_max_ of 294 ng/mL and declined in a multiphasic manner with a similar *t*_1/2_ of 8.76 hours. No adverse clinical signs were observed in mice during the 24 hours of the in vivo PK studies.

We also tested for possible adverse actions of M1i-124 on cytochrome P450 (CYP) enzymes that might limit its use in vivo. The CYP family of enzymes are responsible for phase I metabolism of many drugs and endogenous substances, and their inhibition can lead to unanticipated toxicities. M1i-124 exhibited an IC_50_ greater than 10 μM against all CYP enzymes tested, indicating that the compound is a weak inhibitor or non-inhibitor of the major CYPs involved in drug metabolism (Figure 7C). To further test for potential off-target effects of M1i-124, we conducted an in vitro safety pharmacology profiling screen that measures the ability of a candidate compound to inhibit 78 key GPCRs, ion channels, nuclear hormone receptors, neurotransmitter transporters, kinases, and non-kinase enzymes. Notably, the EC_50_ or IC_50_ for M1i-124–dependent inhibition was greater than 10 μM for the most critical targets in the panel, including COX1/2, PDE4, kinases (INSR, LCK, ROCK1, VEGFR2), and hERG (Supplemental Table 2).

We next sought to characterize potential in vivo toxicities of M1i-124. First, using WT C57BL/6 mice, we compared lymphocyte populations within the spleens of mice treated with M1i-124 or vehicle control for 12 days. We found no difference in the percentage of total lymphocytes within the spleen or the percentage of specific lymphocyte populations including cytotoxic T cells, helper T cells, regulatory T cells, B cells, or natural killer cells (Supplemental Figure 7). Next, to determine the effect of long-term administration of M1i-124, we treated C57BL/6 mice with M1i-124 or vehicle control for 28 days and evaluated for changes in weight, complete blood counts, and liver function tests. Long-term treatment of WT mice with M1i-124 did not result in a statistically significant change in weight (Figure 7D) or blood counts (white blood cell count, hemoglobin, or platelet count) (Figure 7E) compared with control. There was a decrease in albumin in M1i-124–treated mice, while, reassuringly, M1i-124 did not result in a change in alanine transaminase or total bilirubin (Figure 7F). Likewise, on H&E analysis, there was no appreciable difference in liver histology between control- and M1i-124–treated mice (Figure 7G).

Taken together, the bioavailability, pharmacokinetic, and off-target studies indicate overall favorable drug-like properties for M1i-124. In vivo administration of M1i-124 was well tolerated with minimal toxicity.

### M1i-124 suppresses ABC-DLBCL tumor growth in vivo.

**Figure 8:**
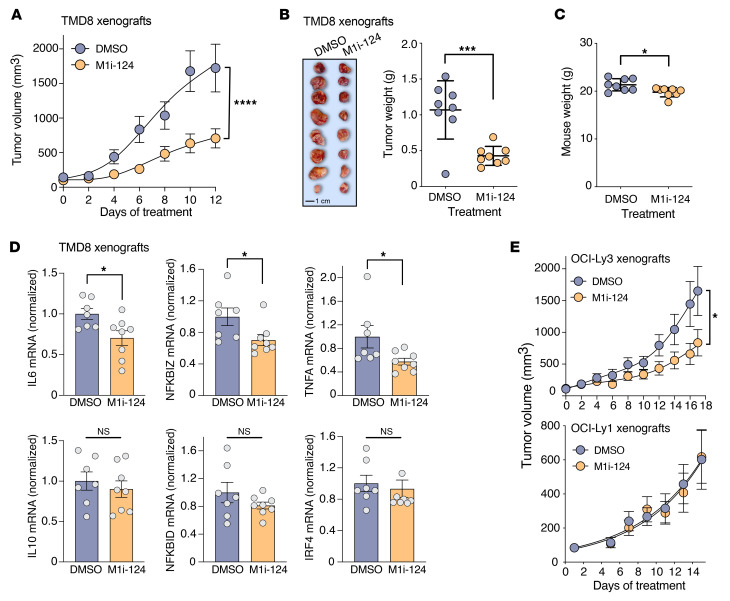
M1i-124 inhibits ABC-DLBCL lymphoma tumor growth in a xenograft mouse model. (**A** and **B**) M1i-124–dependent inhibition of TMD8 xenograft growth in NOD-SCID mice, as assessed by tumor volume (**A**) and terminal tumor weight (**B**) (mean ± SEM; *n* = 8). (**C**) Effect of M1i-124 on mouse weight (mean ± SEM; *n* = 8). (**D**) *IL6*, *IL10*, *IRF4*, *NFKBID*, *NFKBIZ*, and *TNFA* mRNA levels in TDM8 tumor xenografts 24 hours after the final treatment with M1i-124 or DMSO control (mean ± SEM; *n* = 7–8). (**E**) M1i-124–dependent inhibition of OCI-Ly3 (ABC-DLBCL line) versus OCI-Ly1 (GCB-DLBCL line) xenograft growth in NSG mice, as assessed by tumor volume (mean ± SEM; *n* = 7–8). For all panels, statistical analyses were performed using unpaired *t* test. *P < 0.05, ***P < 0.001, ****P < 0.0001.

After establishing the overall favorable drug-like properties of M1i-124, we evaluated the in vivo efficacy of the compound in reducing ABC-DLBCL lymphoma growth. TMD8 xenografts were established in female NOD-SCID mice, which were then randomized to receive daily i.p. injection of either M1i-124 or DMSO control for 12 days. The growth of the TMD8 tumor xenografts, tracked by tumor volume and terminal tumor weight, was significantly suppressed by M1i-124 treatment as compared with DMSO (Figure 8, A and B). While mice treated with M1i-124 showed slightly lower body weights than control-treated mice at the conclusion of the experiment (Figure 8C), this difference is likely accounted for by the significant reduction in tumor size in mice treated with the M1i-124 compound. No abnormal clinical signs or indicators of adverse reaction were observed during the course of treatment, and comprehensive microscopic evaluation of major organs including heart, lung, liver, adrenal, and kidney from both DMSO- and M1i-124–treated mice showed no signs of histologic abnormality (Supplemental Figure 8).

To complement studies of tumor growth inhibition, we isolated RNA from TMD8 tumor xenografts and tested the in vivo effect of M1i-124 on CBM-dependent signaling. We found that tumors harvested from mice 24 hours after they received their last dose of M1i-124 demonstrated a significant decrease in *IL6* mRNA level compared with tumors from mice receiving DMSO (Figure 8D). *IL10* mRNA levels were not affected at this time point (Figure 8D); this is consistent with a previous report indicating that the MALT1 protease inhibitor, compound 3, has only a short-term effect on *IL10* mRNA levels in vivo, reducing levels within tumors at 6 hours, which then rebound 12–24 hours after administration (22). M1i-124 treatment did reduce tumor levels of certain other MALT1-dependent NF-κB target genes at the 24-hour time point, including *NFKBIZ* and *TNFA*, while having no effect on genes including *IRF4* and *NFKBID* (Figure 8D).

To test M1i-124 in a second in vivo ABC-DLBCL model, we established OCI-Ly3 xenografts in NSG mice, as described previously (40), and randomized mice to receive daily i.p. injections of either M1i-124 or DMSO. As with the TMD8 model, we again observed a significant decrease in tumor growth in mice receiving M1i-124 as compared with DMSO control (Figure 8E). Finally, to confirm in vivo specificity of M1i-124 for ABC-DLBCL versus GCB-DLBCL tumors, we similarly established OCI-Ly1 (GCB-DLBCL) xenografts in NSG mice and tested the response to M1i-124. In contrast to what we observed with the ABC-DLBCL models, results showed no effect of M1i-124 on OCI-Ly1 xenograft growth (Figure 8E).

## Discussion

In this study, we describe the identification of M1i-124 and the derivative M1i-124d1, potentially first-in-class BCL10-MALT1 small-molecule PPI inhibitors that show efficacy in inhibiting both MALT1 protease and scaffolding functions and in selectively suppressing the growth and survival of MALT1-dependent ABC-DLBCL cells. We demonstrate that in addition to inhibiting the BCL10-MALT1 interaction, M1i-124 potently drives loss of MALT1 and BCL10 proteins within cells, an effect that is likely a consequence of its ability to disrupt self-stabilizing BCL10-MALT1 complexes. When tested in vivo, M1i-124 effectively abrogates growth of ABC-DLBCL xenografts and reduces expression of NF-κB target genes within xenograft tumors. Importantly, M1i-124 shows promising drug-like properties that can be improved upon through a systematic process of compound optimization.

**Figure 9:**
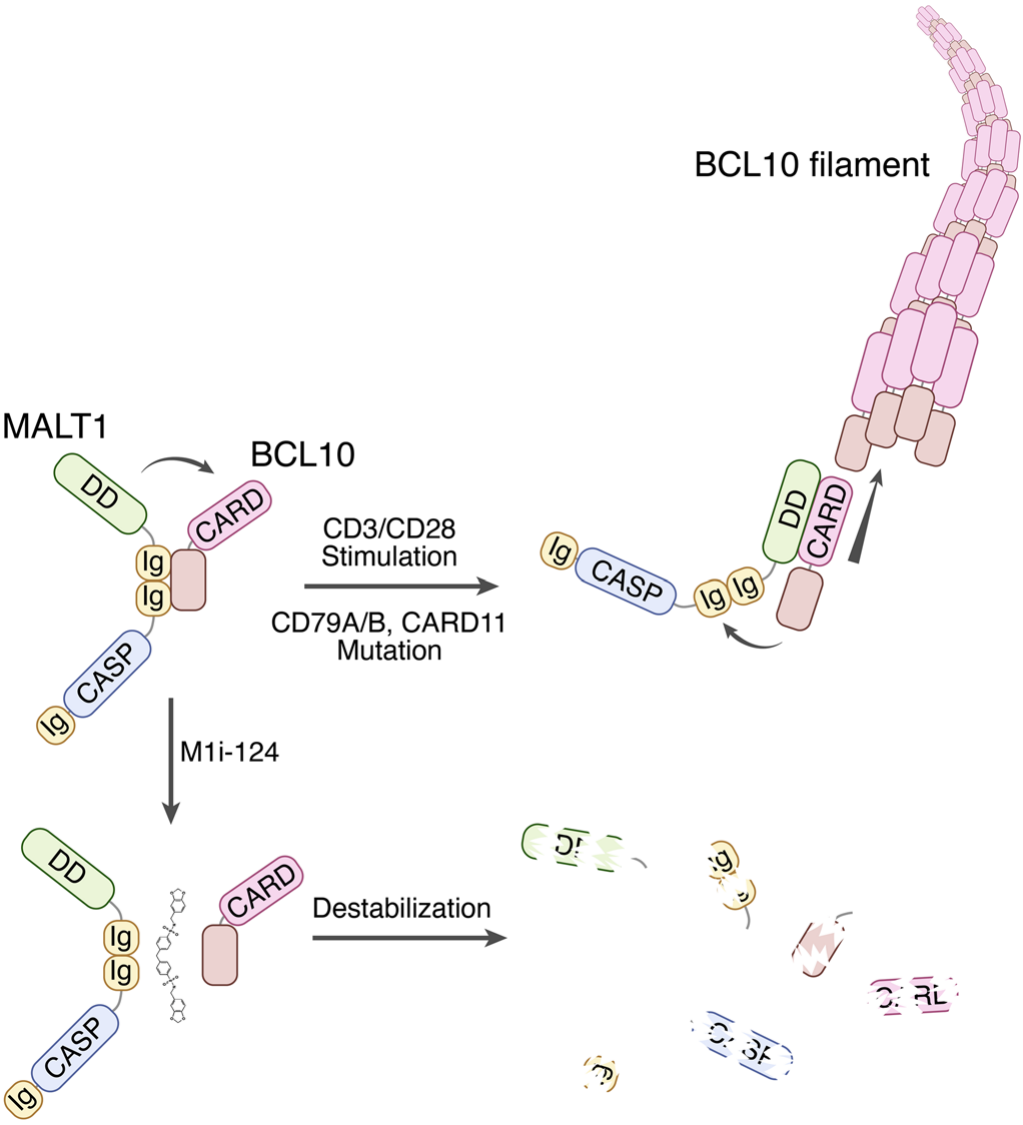
Model for BCL10-MALT1 interaction and the role of M1i-124 in disrupting CBM assembly. This model proposes that the nature of the interaction between BCL10 and MALT1 differs depending on the state of cell activation. In unstimulated lymphocytes, an interaction between the MALT1 tandem Ig domains and the BCL10 C-terminus is responsible for maintaining dimeric complexes. Upon antigen receptor stimulation, or in cells harboring mutations that cause constitutive stimulation, the BCL10-MALT1 dimers undergo a conformational shift resulting in a repositioning of the interaction as the proteins are inserted into a growing CBM filament. In this setting, a MALT1 DD–BCL10 CARD interaction is responsible for maintaining the oligomeric complex. Treatment of cells with M1i-124 causes disruption of the resting dimeric complexes and destabilization of the BCL10 and MALT1 proteins. With time, even in ABC-DLBCL cells harboring activating mutations, the turnover of filaments and lack of dimeric BCL10-MALT1 substrate to generate new filaments result in suppressed CBM activity.

In addition to nominating a new class of small-molecule precision therapeutics, this study is significant in that it also reveals information regarding the intricate mechanisms by which BCL10 and MALT1 bind to one another within the cell, emphasizing the idea that the nature of the interaction likely changes depending on the state of cell activation. One working model that is consistent with our observations and those of previously published studies (13, 33, 34) posits that dimeric BCL10-MALT1 complexes within resting lymphocytes are formed through interaction of BCL10 with MALT1 Ig1-2 domains, but upon antigen stimulation there is a shift in the interaction as BCL10 molecules are inserted into a growing BCL10 filament. This shift may involve a conformational change in MALT1 to release the Ig1-2–dependent interaction so as to establish a new DD-dependent interaction with filamentous BCL10 (Figure 9).

While this manuscript was being prepared, a report was published by the Wu and Melnick laboratories describing their discovery of BCL10 gain-of-function driver mutations in DLBCL (52). Their findings provide additional insight into the role of the MALT1 Ig1-2 region in binding to BCL10. Specifically, the group identified recurrent somatic truncating (nonsense or frameshift) mutations in the C-terminal region of BCL10. They then characterized one truncated BCL10 protein that is generated as a consequence of an E140X (premature STOP) mutation and found that it exhibits loss of basal MALT1 binding in comparison with WT BCL10. This observation led them to investigate the contribution of the BCL10 C-terminal region to interaction with MALT1 and identified BCL10 residues 165–208 as a binding site for the MALT1 Ig1-2 region. The authors found that, unlike the interaction between BCL10 CARD and MALT1 DD that occurs in the setting of BCL10 filaments, the interaction between this BCL10 C-terminal site and MALT1 Ig1-2 occurs between monomeric species, consistent with our observation that MALT1 Ig1-2 binds to the non-oligomerizing BCL10 E53R mutant and with the model depicted in Figure 9. An intriguing question that arises from the new work by the Wu and Melnick team is why C-terminal truncation mutations of BCL10 that result in loss of interaction with MALT1 Ig1-2 can act as gain-of-function mutations, while small molecules that disrupt the interaction between WT BCL10 and MALT1 Ig1-2 can inhibit CBM signaling. One possibility is that while the Ig1-2–dependent interaction between MALT1 and BCL10 is necessary to maintain adequate levels of the proteins in the cell, so that the cell is poised for activation, the interaction also provides some level of inhibition to spontaneous BCL10 oligomerization and filament formation, which must be overcome by upstream signal activation. Indeed, the report by the Wu and Melnick labs suggests that the interaction of MALT1 Ig1-2 with the C-terminal region of BCL10 may inhibit BCL10 polymerization (52). A second possible explanation is that the C-terminal truncation mutant of BCL10 loses IKKβ phosphorylation sites that mediate feedback inhibition of CBM functionality (53). Clearly, this fundamental conundrum underscores the point that more work is needed to fully understand the complexities inherent to CBM signalosome assembly and function.

The loss of cellular BCL10 and MALT1 protein content induced by M1i-124, despite compensatory increases in the corresponding mRNAs for these proteins, may represent one of the most important aspects of the action of the M1i-124 compound. Prior reports suggest that the BCL10-MALT1 interaction within a cell stabilizes each of the binding partners (36, 37), so that inhibiting the interaction via M1i-124 could lead to induced degradation of both proteins. This effect likely explains why the IC_50_ values for cell-based readouts of M1i-124 action are more than 10-fold lower than the IC_50_ for the cell-free ELISA-based readout used to measure BCL10-MALT1 binding inhibition. Loss of MALT1 protein similarly occurs after treatment of cells with MALT1-directed proteolysis-targeting chimera (PROTAC) compounds (54–56). For PROTAC compounds, cellular EC_50_ values are typically lower than the intrinsic *K_D_*, since the efficacy of these agents does not exclusively depend on the equilibrium level of target occupancy (57). While M1i-124 is not designed to specifically engage the ubiquitin-proteasome system for targeted MALT1 degradation, it may act in a manner similar to that of PROTAC compounds by inducing degradation of BCL10 and MALT1. Additionally, a recent report described a BCL10 peptide inhibitor that targets the BCL10 self-association interface and disrupts BCL10 polymerization. This peptide has been shown to induce BCL10 protein destabilization and suppress the growth of ABC-DLBCL tumors in vivo (58). Together with this recent report, our findings emphasize the important role protein interactions play in maintaining the stability of CBM protein components.

In addition to disrupting the BCL10-MALT1 interaction and inducing protein destabilization, another possible explanation for the higher cellular potency of M1i-124 is that the compound may interfere with the conformational changes of MALT1 that are required for assembly of the filamentous CBM complex. Such an effect would not be reflected in the IC_50_ values obtained using the cell-free ELISA-based platform that measures purified recombinant MALT1 binding to immobilized BCL10. As noted above, the Wu and Melnick labs suggested that the interaction of MALT1 Ig1-2 with the C-terminal region of BCL10 may inhibit BCL10 polymerization (52), so it is tempting to speculate that by binding to the MALT1 Ig1-2 domains, M1i-124 may somehow “lock” MALT1 into a conformation that prevents the formation of CBM filaments. Additional studies will be needed to test for effects of M1i-124 on MALT1 conformation and CBM filament formation.

Finally, the IC_50_ for the MALT1 protease inhibitor MI-2 was also reported to be significantly lower when measured using cell-based assays than when measured in a cell-free enzymatic assay system (23). The authors of that study proposed that this difference in IC_50_ is likely explained by the irreversible binding of MI-2 to MALT1 and/or the intracellular accumulation of MI-2. Together, findings from the study of both M1i-124 and MI-2 indicate that cell-free studies of MALT1 inhibitors may yield IC_50_ values that underestimate the potential potency of these compounds when used in cell-based, biologic assays, albeit for different reasons depending on their putative mechanisms of action.

About 40% of patients with DLBCL remain refractory or relapse after the standard R-CHOP immunochemotherapy. Over the last 20 years, nearly every phase III clinical trial testing various targeted therapies combined with R-CHOP has failed to meet its primary endpoint to improve event-free or progression-free survival. Recent efforts to refine the cell-of-origin and genomic classification for DLBCL subtypes may provide a basis for improved risk stratification and response to new targeted therapies, including MALT1 inhibitors (4–6). For example, ABC-DLBCL tumors driven by specific activating mutations in BCL10 are not sensitive to inhibitors of Bruton’s tyrosine kinase (BTK) — an expected finding since BTK operates upstream of the CBM complex — but do show enhanced sensitivity to MALT1 protease inhibition (52). Although existing MALT1 protease inhibitors have been shown to effectively reduce ABC-DLBCL tumor growth in mouse xenograft models, these inhibitors only abrogate the protease arm of MALT1 action and do not inhibit MALT1 scaffolding function so that the mechanisms for direct IKK activation and induced IκB degradation remain unobstructed. In addition, concerns have been raised regarding the risk of potential autoimmune/inflammatory toxicities for these compounds. Germline inactivation of MALT1 protease activity, in the setting of retained MALT1 scaffolding function, has been shown to induce an imbalance in the ratio of regulatory T cells (Tregs) to effector T cells (Teffs), resulting in a severe autoimmune phenotype (25–28). Similarly, pharmacologic treatment with MALT1 protease inhibitor in rat and dog models has also been shown to lead to rapid reduction in Tregs and expansion of Teffs, with progressive development of severe multiorgan inflammation (29). In contrast, treatment with compounds like M1i-124, which block both the protease and scaffolding activities of MALT1, could be expected to avoid the potential autoimmune toxicity associated with unbalanced T cell subsets.

In addition to ABC-DLBCL, MALT1-dependent activation of the NF-κB transcription factor drives the pathogenesis of multiple other B and T cell malignancies, including subsets of mantle cell lymphoma, adult T cell leukemia, and chronic lymphocytic leukemia with chronic B cell receptor signaling (7, 59–61). Given the critical role that MALT1 plays in T cell activation and regulation, there is also emerging interest in the use of MALT1 inhibitors in autoimmune and inflammatory diseases, including psoriasis (62–65), colitis (25, 66–68), rheumatoid arthritis (69), and autoimmune encephalitis/multiple sclerosis (25, 70, 71). Because M1i-124 inhibits both MALT1 protease and scaffolding functions, we might expect a more balanced inhibition of both Tregs and Teffs, making these compounds particularly promising for the long-term treatment of autoimmune/inflammatory diseases.

In summary, we believe M1i-124 and M1i-124d1 are first-in-class BCL10-MALT1 PPI inhibitors that demonstrate preclinical efficacy in selectively suppressing the proliferation and survival of MALT1-dependent ABC-DLBCL. While there are challenges associated with developing small-molecule drugs that elicit beneficial effects by inhibiting protein-protein interaction, notable successes are emerging, and the category of clinically deployed PPI inhibitor pharmaceuticals is expanding. As one example, ABT-199 (venetoclax), which inhibits the interaction of BCL-2 with downstream pro-apoptotic proteins, represents the first small-molecule PPI inhibitor approved for the treatment of chronic lymphocytic leukemia, small lymphocytic lymphoma, and acute myeloid leukemia (72). Early in vivo PK and off-target liability studies demonstrate an overall favorable safety profile for M1i-124, indicating that it can be advanced for further optimization and clinical development. Finally, this study suggests that small-molecule targeting of the BCL10-MALT1 interaction holds promise as a therapeutic strategy for an even broader range of B and T cell malignancies that, like ABC-DLBCL, also depend on constitutive MALT1 signaling, as well as for selected autoimmune disorders.

## Methods

Additional methods are described in Supplemental Methods.

### Sex as a biologic variable.

Xenograft experiments using DLBCL models were performed in female mouse hosts owing to more consistent engraftment, but the response of fully engrafted DLBCL tumors to drug treatments is typically similar in male mice. Toxicity studies of M1i-124 were performed using an equal number of male and female C57BL/6 mice.

### In silico screening and compound identification.

The LibDock module from Discovery Studio 3.5 was used to perform a structure-guided in silico screen of 3 million compounds to identify candidate molecules that could potentially fit within the identified groove of MALT1(Ig1-2) (PDB ID: 3K0W). Details are provided in Supplemental Methods.

### Surface plasmon resonance.

Surface plasmon resonance (SPR) experiments were performed on a Biacore 2000 system (Cytiva). Full-length GST-BCL10 (Abnova, catalog H00008915-P01) was immobilized onto a gold sensor chip surface, and analyte solution containing MALT1(DD+Ig1-2), MALT1(DD), or MALT1(Ig1-2) purified protein was injected over the surface through a series of flow cells (see Supplemental Methods for details on protein purification). The binding kinetic parameters were obtained using the Langmuir model for global fitting with 1:1 binding ratio where one ligand molecule interacts with one analyte molecule. The equilibrium dissociation constant (*K_D_*) of M1i-124 to full-length GST-tagged MALT1 (Novus, catalog H00010892-P01) protein was determined by Creative Biolabs using Biacore T200. Full-length MALT1 protein was immobilized onto CM5 sensor chip surface, and M1i-124 was injected at concentrations of 0, 12.5, 25, 50, 100, and 200 μM. The steady-state affinity model was used to determine the *K_D_*.

### ELISA-based protein-protein interaction assay.

A 96-well ELISA was employed, whereby His-tagged full-length BCL10 protein was immobilized in wells and exposed to GST-tagged recombinant human full-length MALT1 protein in solution, followed by detection with anti-MALT1 antibody. Details are provided in Supplemental Methods.

### Evaluation of MALT1 protease and scaffolding functions.

Western blot analysis of RelB and N4BP1 cleavage was used to test the effect of small molecules on MALT1 protease activity. To this end, 3 million Jurkat T cells were pretreated with compounds for 24 hours in serum-free media, and then 5 μM MG-132 was added for 1 hour before stimulation with CD3/CD28 (Stemcell Technologies, catalog 10971), and lysed in RIPA buffer (Millipore, catalog 20-188) with protease and phosphatase inhibitor cocktail (Pierce, catalog 78446). IKKα/β phosphorylation and IκBα phosphorylation were used as measures of MALT1 scaffolding activity, while ERK1/2 phosphorylation was used as a control. Two million Jurkat T cells were treated with 1 μM M1i-124, 1 μM M1i-124d1, or DMSO in serum-free media for 16 hours, stimulated with CD3/CD28 for 0, 5, 10, or 20 minutes, and then immediately lysed in RIPA buffer with protease and phosphatase inhibitors. For experiments evaluating RelB cleavage and phosphorylation of IκBα in TMD8 cells, cells were treated with compounds for 24 hours in media containing 5% FBS. For RelB Western blot analysis, 5 μM MG-132 (Cell Signaling Technology, catalog 2194s) was added 4 hours before lysis in RIPA lysis buffer with protease/phosphatase inhibitors.

### Xenograft models of DLBCL.

TMD8 cells were engrafted into 8-week-old female NOD.CB17-Prkdc^scid^/J (NOD-SCID) mice from The Jackson Laboratory by subcutaneous injection of 10 × 10^6^ cells resuspended in 50% Cultrex Basement Membrane Extract (R&D Systems, catalog 3632-010-02) into the right flank. OCI-Ly3 cells were engrafted into 5-week-old female NOD.Cg-Prkdc^scid^ Il2rg^tm1Wjl^/SzJ (NSG) mice from The Jackson Laboratory by subcutaneous injection of 2.5 × 10^6^ cells resuspended in Cultrex as above. OCI-Ly1 cells were engrafted into 6.5-week-old female NSG mice from The Jackson Laboratory by subcutaneous injection of 20 × 10^6^ cells resuspended in Cultrex as above. For all xenograft models, when tumors reached an average volume of 100 mm^3^, mice were randomized to receive M1i-124 (50 mg/kg i.p. per day), or equivalent volume of DMSO vehicle (50 μL i.p. per day). M1i-124 dosing was based on the results of PK studies indicating that 50 mg/kg/d would provide sufficient drug exposure. Tumor size was measured 3 times a week using a caliper and calculated using the formula (smallest diameter^2^ × largest diameter)/2. Mice were sacrificed 24 hours after the last injection. Mice were sacrificed when the largest tumors reached 2,500 mm^3^. For TMD8 xenograft experiments, tumors and various organs, including lung, heart, liver, and kidney, were collected and fixed in 10% buffered formalin, embedded in FFPE blocks, sectioned, and stained with H&E. Sections were evaluated by a board-certified pathologist.

### Statistics.

All values are represented as mean ± SEM. Data were analyzed and statistics performed using GraphPad Prism software. Two-tailed Student’s *t* test was used to compare 2 groups, and ANOVA test was used to compare 3 or more groups. Dose-response curves were fitted using the following equation: *Y* = bottom + (top – bottom)/1 + 10^(X^^–^^logIC50)^, with the top and bottom representing the plateaus in the units of the *y* axis. In vivo tumor growth curves were evaluated using 2-way ANOVA and Šidák’s multiple-comparison tests.

### Study approval.

All experiments involving animals were approved by the University of Pittsburgh IACUC (protocol 19126343).

### Data availability.

Values for all data points in graphs are reported in the Supporting Data Values file.

## Author contributions

HK and LMM designed research studies, conducted experiments, analyzed data, and wrote the manuscript. HK and LMM are co–first authors, with HK listed first as she initiated the project. JC designed research studies, conducted experiments, and analyzed data related to the in vivo models. MS and LRK designed research studies, conducted experiments, and analyzed data. DH designed experiments and created key reagents. JHA designed and performed key experiments. MJM, AM, EA, and ZNC designed research studies, conducted experiments, and analyzed data for studies of the binding of MALT1 fragments to BCL10 by SPR. HBK, ML, PE, and MT acquired data. BBC designed and conducted the in silico drug screen. PCL and LMML are the principal investigators of the laboratory where they supervised the development of this project in its entirety, procured funding to support the work, and led the construction of the manuscript. All authors read and approved the final manuscript.

## Supplementary Material

Supplemental data

Unedited blot and gel images

Supporting data values

## Version 1 04/15/2025

Electronic publication
