# Supplementary material for: A small-molecule inhibitor of BCL10-MALT1 interaction abrogates progression of diffuse large B cell lymphoma
Source: J Clin Invest. 2025 Apr 15;135(8):e164573. doi: 10.1172/JCI164573 (PMC11996864; doi:10.1172/JCI164573)
Supplement: Supplemental data [file jci-135-164573-s323.pdf]

Supplemental Materials for

A small molecule inhibitor of BCL10-MALT1 interaction abrogates progression of Diffuse Large B-cell Lymphoma

Heejae Kang,[*,1] Lisa M. Maurer,[*,2] Jing Cheng,[2,3] Mei Smyers,[2,†] Linda Klei,[2] Dong Hu,[1,3] Juliana Hofstatter Azambuja,[2,3] Marcelo J. Murai,[4] Ahmed Mady,[4] Ejaz Ahmad,[4] Matthew Trotta,[2,‡] Hanna B. Klei,[2] Minda Liu,[2,††] Prasanna Ekambaram,[2] Zaneta Nikolovska-Coleska,[4] Bill B. Chen,[5] Linda M. McAllister-Lucas,[†,2,6,7,8] and Peter C. Lucas[†,1,3,6,8]

[1]Department of Pathology and [2]Department of Pediatrics, University of Pittsburgh School of Medicine, Pittsburgh, Pennsylvania, USA. [3]Department of Laboratory Medicine and Pathology, Mayo Clinic, Rochester, Minnesota, USA. [4]Department of Pathology, University of Michigan Medical School, Ann Arbor, Michigan, USA. [5]Department of Medicine, University of Pittsburgh School of Medicine, Pittsburgh, Pennsylvania, USA. [6]UPMC Hillman Cancer Center, Pittsburgh, Pennsylvania, USA. [7]Department of Pediatrics and Adolescent Medicine, Mayo Clinic, Rochester, Minnesota, USA. [8]Mayo Clinic Comprehensive Cancer Center, Rochester, Minnesota, USA.

This pdf file includes:

- Supplemental Materials and Methods
- Supplemental References
- Supplemental Figures 1 – 8
- Supplemental Tables 1 and 2

Supplemental Materials and Methods

Cell lines and reagents. The DLBCL cell lines OCI-Ly1, OCI-Ly3, and OCI-Ly7 were provided by Mark D. Minden (University Health Network, Toronto, Ontario, Canada), TMD8 cell line was provided by Louis Staudt (National Cancer Institute, NIH, Bethesda Maryland), and the BJAB cell line was provided by Mathijs Baens (Katholieke Universiteit Leuven, Leuven, Belgium). Cells were grown in culture as previously described (1). Jurkat T cells, Clone E6-1, were purchased from ATCC and grown in ATCC modified RPMI 1640 (ThermoFisher Scientific, Cat# A1049101) supplemented with 10% fetal bovine serum (FBS) (Cytiva, Cat # SH30396.03). HEK293T cells (CRL-11268) were grown in Dulbecco's Modified Eagle Medium (DMEM) containing GlutaMax (ThermoFisher Scientific, Cat# 10569-010) and supplemented with 10% FBS. All cell lines were maintained at 37°C, 5% CO2. Cell lines underwent regular STR profiling (University of Arizona Genetics Core) and mycoplasma testing (University of Arizona Genetics Core or Lonza, Cat# LT07-118). Stimulation of Jurkat T cells was accomplished by the addition of phorbol 12-myristate 13 acetate (PMA) (5 ng/mL Sigma, Cat# P8139) and ionomycin (0.5μM; EMD Chemicals Millipore, Cat# 407951) or ImmunoCult Human CD3/28 activator (5-10 μL/mL, Stemcell Technologies, Cat# 10971). Mouse splenocytes were stimulated with PMA/ionomycin as above or Dynabeads™ Mouse T-Activator CD3/C28 (ThremoFisher, Cat# 11452D). Mepazine was purchased from EMD Millipore (Cat# 500500).

In silico screening and compound identification. The LibDock module from Discovery Studio 3.5 was used to perform a structure-guided in silico screen of 3 million compounds to identify candidate molecules that could potentially fit within the identified groove of MALT1(Ig1-2) (PDB: 3K0W). Lipinski's rule of five filters were applied to enrich for compounds with drug-like properties. Compounds identified in the screen and the lead compound derivative were purchased from ChemDiv: M1i-124 (K691-0124), M1i-124d1 (K691-0122), C260-1522, C519-2094, C741-0547, A0070495, C200-2344, and C260-1268. Compounds V001-9748 and V024-1168 identified in the in silico drug screen was not available at the time compound screening experiments were performed, though V001-9748 (ChemDiv) was purchased later as a control for some experiments. Docking of M1i-124 onto Ig1-2 was achieved using LibDock, and additional visualizations were performed with PyMOL (Schrodinger, Inc).

Purification of proteins for SPR. Recombinant human MALT1 constructs and human BCL10 protein were purified after expression in *E. coli* following standard protocols. Briefly, MBP-MALT1(DD+Ig1-2)(aa 1-326), GB-MALT1(DD)(aa 29-124), SUMO-MALT1(Ig1-2)(aa 128-326), and full-length BCL10 were expressed in *E. coli* strain BL21(DE3). Proteins were purified by affinity chromatography (HisTrap FF, Cytiva), followed by ion exchange chromatography (HiTrap Q, Cytiva). Size exclusion chromatography (Superdex200 10/300, Cytiva) was utilized for the final purification step of full-length BCL10. Before use in the SPR assay, tags were cleaved by TEV and U1p1 proteases for MBP-MALT1(DD+Ig1-2) and SUMO-MALT1(Ig1-2), respectively, and removed by subtractive affinity chromatography.

Plasmids. Plasmids for transfection were verified with Sanger Sequencing. Unless otherwise indicated, plasmids were made by subcloning cDNA fragments from constructs described in previous publications, as noted, and/or site directed mutagenesis. Plasmids in the pcDNA3 or pcDNA3.1 backbone include HA-MALT1(isoform B) (2), HA-MALT1(DD+Ig1-2) (aa 1-330), HA-MALT1(DD) (aa 1-139), HA-MALT1(Ig1-2) (aa 128-330) (Vector Builder), HA-MALT1(Ig1-2+CTD) (aa 128-813) (Vector Builder), HA-MALT1(Ig1-2) F187A, HA-MALT1(Ig1-2) V189R, HA-MALT1(Ig1-2) W203_Q205delinsAAA (Vector Builder), HA-MALT1(Ig1-2) H257_Q259delinsAAA (Vector Builder), HA-MALT1(Ig1-2) P254_P256delinsAAA (Vector Builder), HA-MALT1(Ig1-2) H269_T271delinsAAA (Vector Builder), HA-MALT1(Ig1-2) K272_L274delinsAAA (Vector Builder), HA-MALT1 V189R (Vector Builder), HA-MALT1 V81R, HA-BCL10 (GeneCopoeia, Cat# M0769-M07), HA-BCL10 E53R, Myc-BCL10 (previously called CIPER) (3), Myc-BCL10 E53R, Myc-RICK (4), Myc-MAVS (subcloned from FLAG-MAVS (5)), and empty vector pCDNA3.1HA as a control.

ELISA-based protein-protein interaction assay. On a high-binding 96-well ELISA microplate (Greiner Cat# 655061), 100 ng of His-tagged recombinant human full-length BCL10 protein (Novus Cat# NBP1-99064) or in 100 µl PBS was allowed to adhere overnight at 4°C. The plate was blocked with 5% BSA in PBS for 1 hour at room temperature. After washing twice with PBS-T, a mixture of 100 ng of GST-tagged recombinant human full-length MALT1 protein (Novus Biologicals Cat# H00010892-P01) and compound that was preincubated for 1 hour in 100 µl PBS was added to the plate for 1 hour at room temperature. After washing three times with PBS-T, anti-MALT1 antibody (Cell Signaling Technology, Cat# 2494) in 5% BSA in PBS was added at 1:1000 dilution for 1 hour at room temperature. After washing three times with PBS-T, HRP-conjugated secondary antibody in 5% BSA in PBS was added at 1:5000 dilution for 1 hour at room temperature. After washing three times with PBS-T, 100 µl of 3,3',5,5'-tetramethylbenzidine (TMB) substrate solution (Thermo Scientific, Cat# 34028) was added for up to 15 minutes, and 100 µl of 2 M sulfuric acid was added to stop the reaction. The plate was read at 450 nm using a spectrophotometer (Molecular Devices SpectraMax i3). The MyoD1/ID ELISA was performed as above except that 100 ng of recombinant human His-tagged MyoD1 (Proteintech, Cat# Ag13186) was plated overnight at 4°C prior to the addition of 100 ng of recombinant human GST-ID1 (Proteintech, Cat# Ag13359) preincubated to with compounds. The anti-GST antibody (clone 26H1, Cell signaling Technology, Cat #2624) was used to detect bound ID1.

Cytokine ELISAs. Cytokines in cell culture supernatants were measured 20-24h after treatment with 0, 0.01, 0.03, 0.1, 0.3, 1, or 3 µM of compounds using the following ELISA kits according to the manufacturer's instructions: Human IL-2 ELISA (BioLegend Cat# 431804), Human IL-6 ELISA (BioLegend Cat# 430504), and Human IL-10 ELISA (BioLegend Cat# 430604). TMD8 and OCI-Ly3 cells were treated with specific doses of compounds in serum-free media for 20 hours. Jurkat T cells were pretreated with specific doses of compounds for 8 hours in serum-free media, then stimulated with PMA/ionomycin for 16 hours prior to the collection of supernatants. Mouse splenocytes were isolated from spleens of C57/BL6 mice by passing spleens through a 70 micron filter followed by red blood cell lysis using ACK lysing buffer (Quality Biologics, Cat# 118-156-101). Splenocytes were cultured overnight in phenol free RPMI with 10% FBS, and with M1i-124 (1 µM) or mepazine (5 µM) prior to stimulation with PMA/ionomycin or Dynabeads^{TM} Mouse T-Activator CD3/C28. Supernatant was collected at 24 hours and mouse IL-2 levels were analyzed via ELISA (R&D Systems, Cat# DY402-05).

Transfection. HEK293T or HepG2 BCL10$^{-/-}$ cells (used only in Supplemental Figure 1 where indicated) were transiently transfected at approximately 80% confluence using Lipofectamine 3000 (ThermoFisher, Cat# L3000015) per manufacture instructions. Cells were lysed in buffer (150 mM NaCl, 2 mM EDTA, 10% glycerol, 1% Nonidet P-40, 20 mM Tris Hcl, pH 7.4) containing protease and phosphatase inhibitors (Thermo Scientific, Cat#78446) at 24 hours for HEK293T cells or 48 hours for HepG2 BCL10$^{-/-}$ cells.

Immunoprecipitation. Cells were rinsed in PBS and lysed in buffer (150 mM NaCl, 2 mM EDTA, 10% glycerol, 1% Nonidet P-40, 20 mM Tris HCl, pH 7.4) containing protease and phosphatase inhibitors (Thermo Scientific, Cat#78446). Supernatants were incubated with anti-Myc tag agarose beads (Takara, Cat# 631208) or anti-HA tag matrix (Roche, Cat# 11815016001) overnight at 4°C with gentle rotation. Beads were then pelleted at 1000 rpm for 2 min and washed 3-4 times with buffer. Antibody-protein conjugates were removed from beads by boiling for ~5-8 minutes in 2x Laemmli sample buffer (Bio-Rad Laboratories, Cat# 161073) containing 2-mercaptoethanol, and samples were then subjected to SDS-PAGE and immunoblotting.

Antibodies for western blots. The HRP-conjugated anti-HA (clone 3F10, Cat# 12013819001) and anti-HA (clone 3F10, Cat# 11867423001) antibodies were purchased from Sigma. The anti-phospho-IKKα(Ser176)/ IKKβ(Ser177) (clone C84E11, Cat# 2078), rabbit anti-GAPDH (clone D16H11, Cat# 5174), anti-MALT1 (Cat #2494), anti-Myc tag (Cat# 2272), anti-RelB (clone C1E4, Cat# 4922), anti-p44/42 MAPK (Erk1/2) (Cat# 9102), anti-phospho-p44/42 MAPK (Erk1/2) (Thr202/Tyr204) (Cat# 9101), anti-phospho-IκB Ser32/Ser36 (clone 5A5, Cat# 9246) antibodies were purchased from Cell Signaling Technology. The mouse anti-GAPDH (clone 6C5, Cat# sc-32233), anti-MALT1 (clone B-12, Cat# sc-4677), anti-BCL10 (clone H-197, Cat# sc-5611), and anti-IKKα/β (clone H-470, Cat# sc-7607) antibodies were purchased from Santa Cruz. The anti-N4BP1 antibody (Cat# CPA2415) was purchased from Cohesion Biosciences. The anti-mouse (Cat# W4021) and anti-rabbit (Cat# W4011) HRP-conjugated secondaries were purchased from Promega. The anti-Rat HRP-conjugated secondary was purchased from Cell Signaling Technology (Cat# 7077).

qRT-PCR. Jurkat T cells grown in serum free media for 4 hours with indicated inhibitors prior to stimulation with PMA/Ionomycin for 4 hours. TMD8 cells were treated with small molecule inhibitors for 24 with cultures conditions described above except that FBS was 5%. OCI-Ly3 were treated with inhibitors in serum free media for 24 hours. Total RNA extraction from cell lines was performed using RNeasy Plus Mini Kit (Qiagen, Cat# 74136). Total RNA extraction from the TMD8 tumor xenografts was performed using 80% TRIzol Reagent (Fisher Scientific, Cat# 15596026) and 20% Chloroform for tissue solubilization. After centrifugation, the supernatant was mixed with equal volume of 70% ETOH followed by RNA isolation using the RNeasy Plus Mini Kit. RNA was transcribed into cDNA using High-Capacity cDNA Reverse Transcription Kit (Applied Biosystems, Cat# 4368814) according to manufacturer's instructions. RT-PCR was performed using TaqMan Universal PCR Master Mix (Applied Biosystems, Cat# 4304437) and run on a StepOnePlus Real-Time PCR machine (Applied Biosystems). The following probes from ThermoFisher Scientific were used for qRT-PCR: *IL2* TaqMan probe Hs00174114, *IL6* TaqMan probe Hs00174131, *IL10* TaqMan probe Hs00961622, *IRF4* TaqMan Hs01056534, *TNFα* TaqMan

Hs00443260, *NFKBID* TaqMan Hs1071953, *NFKBIZ* TaqMan Hs00230071, and *GAPDH* Taqman Hs02786624_g1.

Cell proliferation, viability, and apoptosis assays. Starting with ~20,000 DLBCL cells in 5% FBS at day 0, cell number was counted using the trypan blue dye exclusion method with Vi-CELL cell viability analyzer (Beckman Coulter) at days 7, 10, and 12 of treatment with 1 μM M1i-124, 1 μM M1i-124d1, 5 μM mepazine, or DMSO control. CellTrace Violet reagent (ThermoFisher, Cat# C34571) was used as a cell division tracking dye for OCI-Ly1, OCI-Ly3, and TMD8 cells treated with 1 μM of compound M1i-124 or DMSO control, where the intensity of the staining decreases with each round of cell division. At days 0 and 6 after staining of DMSO- and compound-treated cells, CellTrace Violet fluorescence was measured on a BD LSR II instrument and Mean Fluorescent Intensity (MFI) was analyzed by FlowJo (version 10.8.0, BD). CellTiter-glo luminescence assay (Promega, Cat# G7570) was used to quantify ATP levels as a surrogate marker of metabolic viability in DLBCL cells treated with compounds for 6-8 days. Annexin V Alexa 488 (ThermoFisher Scientific, Cat# A13201) and SYTOX Blue Dead Cell (ThermoFisher Scientific, Cat# S34857) stains were used to detect apoptosis in DLBCL cells after treatment with 1 μM compounds for 8 days in TMD8 and OCI-Ly1 and for 5 days in OCI-Ly3 cells, then analyzed with flow cytometry. The percentages of cells in early and late apoptosis were combined for quantification. For CellTrace Violet, CellTiter-glo, and Annexin V apoptosis assays, cell were cultured in media with 50% of the usual serum.

Human T cell apoptosis. Human CD4/CD8 T cells were isolated from blood using Pan T Cell Isolation Kit (Miltenyi Biotec, Cat# 130-096-535), IRB exemption STUDY21080109. T cells were incubated with Dynabeads™ Human T-Activator CD3/CD28 (Thermo Fisher Scientific, Cat# 11131D) for 72 hours after which cells were grown in RPMI containing 10% FBS and IL-2 (Peprotech, Cat# 200-02). T cells were treated with M1i-124 (1 μM) or DMSO control with media change/drug treatment every 2-4 days. Apoptosis was evaluated on day 6 as described previously except that annexin V 647 was utilized (Invitrogen, A23204). Data is from 3 independent donors.

Pharmacokinetic and cytochrome P450 inhibition studies. Studies were performed with the assistance of Touchstone Biosciences (Plymouth Meeting, Pennsylvania). For pharmacokinetic bioanalysis, male CD-1 mice were fasted overnight before being dosed with M1i-124 via oral gavage (20mg/kg), intraperitoneal injection in the lower right quadrant of the abdomen (20 mg/kg), or intravenous tail vein injection (5 mg/kg). All blood samples were taken via tail vein at 5, 15, and 30 minutes, and 1, 2, 4, 6, 8, and 24 hours after compound administration. The plasma concentration of the compound was determined using LC-MS/MS analysis. For reversible CYP inhibition studies, different concentrations of M1i-124 were added to 0.2 mg/ml pooled, mixed-gender human liver microsomes (HLM). Each CYP450 isoform-specific probe substrate (CYP1A2, CYP2C9, CYP2C19, CYP2D6, and CYP3A4) was incubated individually with HLM and M1i-124 or corresponding positive control inhibitor (Phenacetin, Tolbutamaide, S-Mephenytoin, Dextromethorphan, Midazolam, and Testosterone). Samples were analyzed for metabolites of the probe substrates by LC-MS/MS at each of the test compound concentrations. A decrease in the formation of the metabolites compared to vehicle control was used to calculate the IC50.

Long-term in vivo toxicity experiments. WT C57/BL6 (The Jackson Laboratory) male and female mice were treated daily with 50 mg/kg M1i-124 or vehicle control for 28 days. At the time of sacrifice, blood was obtained for complete blood count, alanine transaminase, albumin, and total bilirubin (Abaxis). Livers from control and M1i-124 treated mice were collected for H&E analysis.

Flow cytometry. Splenocytes were isolated from WT mice after 12 days of treatment with 50 mg/kg M1i-124 or vehicle control. After obtaining a single cell suspension using 70 µM filter, cells were stained with viability dye (Tonbo Biosciences Ghost Red 780, Fisher Healthcare, Cat #50-105-2988). After blocking with mouse BD Fc Block™ (BD Biosciences, Cat# 553141), splenocytes were stained with a panel of antibodies including antibodies from Invitrogen (anti-CD3 APC, Cat# 47-0031-82; anti-CD8 PerCP-Cyanine 5.5, Cat# 45-0081-82; anti-CD25 PE-Cyanine 5.5, Cat# 35-0251-82 and anti-NK1.1 PE-Cyanine 7 Cat#25-5941-82), an antibody from BD Bioscience (anti-CD45 BUV395, Cat# 564279), and antibodies from Biolegend (anti-CD19 BV421, Cat# 115538 and anti-CD4 BV711 Cat# 100557). Samples were run on a 5 laser LSR Fortessa flow cytometer and analyzed with FlowJo software.

1. Cheng J, et al. GRK2 suppresses lymphomagenesis by inhibiting the MALT1 proto-oncoprotein. *J Clin Invest.* 2020;130(2):1036-51.
2. Lucas PC, et al. Bcl10 and MALT1, independent targets of chromosomal translocation in malt lymphoma, cooperate in a novel NF-kappa B signaling pathway. *J Biol Chem.* 2001;276(22):19012-9.
3. Koseki T, et al. CIPER, a novel NF kappaB-activating protein containing a caspase recruitment domain with homology to Herpesvirus-2 protein E10. *J Biol Chem.* 1999;274(15):9955-61.
4. Inohara N, et al. An induced proximity model for NF-kappa B activation in the Nod1/RICK and RIP signaling pathways. *J Biol Chem.* 2000;275(36):27823-31.
5. Seth RB, et al. Identification and characterization of MAVS, a mitochondrial antiviral signaling protein that activates NF-kappaB and IRF 3. *Cell.* 2005;122(5):669-82.

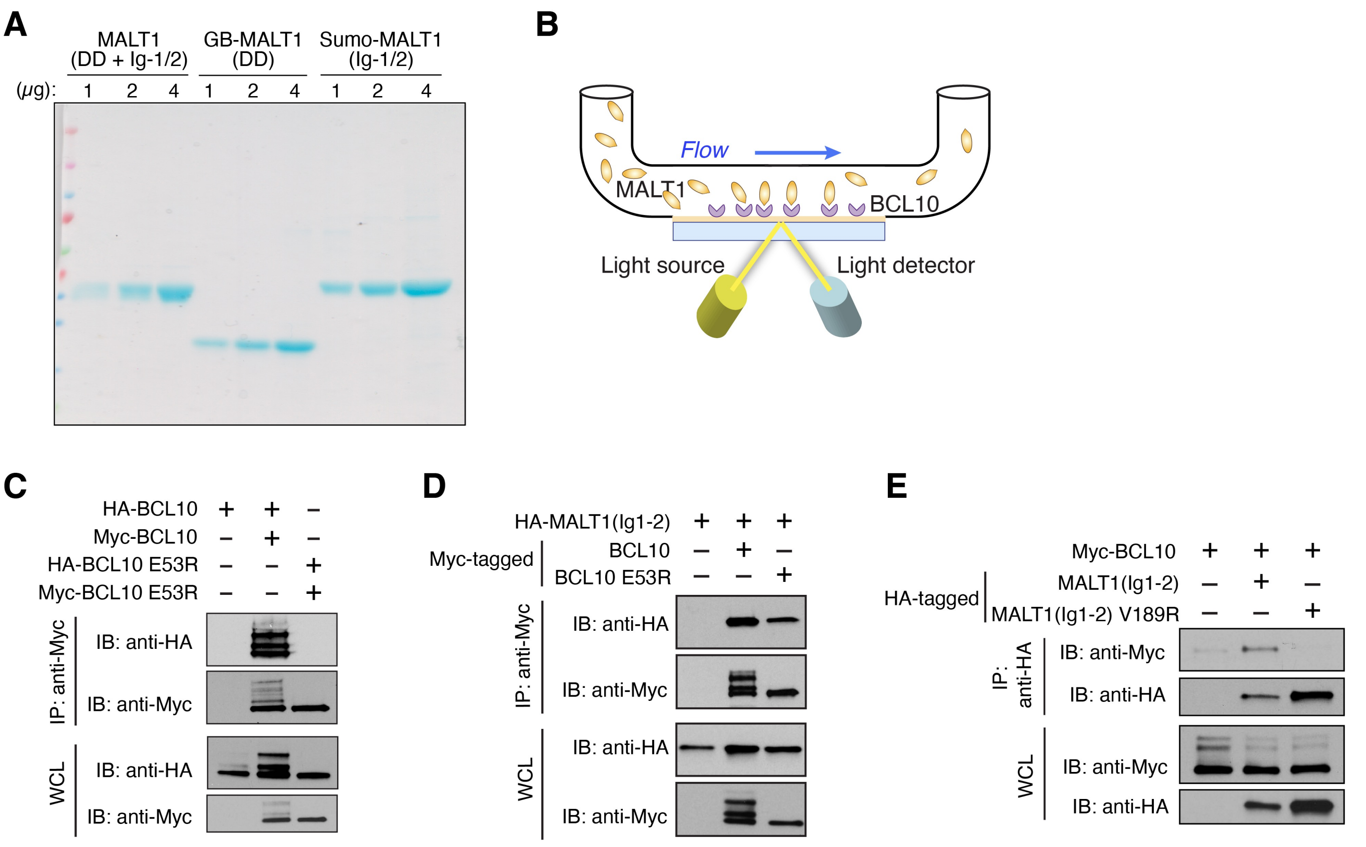

Supplemental Figure 1. MALT1(Ig1-2) binds to BCL10.
(A) Coomassie gel showing purified MALT1 constructs used in the SPR analysis. **(B)** Schematic of SPR assay with immobilized full-length BCL10 and MALT1 fragments in solution. **(C)** Co-IP demonstrating that while wild-type BCL10 self-associates, the E53R mutant is incapable of self-associating. HA- and Myc-tagged proteins were expressed in HEK293T cells prior to lysis and co-IP. **(D)** HA-tagged MALT1(Ig1-2) binds to either wild-type BCL10 or the E53R mutant of BCL10, as demonstrated by co-IP using cells with a BCL10-null (BCL10$^{-/-}$) background. **(E)** Co-IP of HA-tagged MALT1(Ig1-2) or MALT1(Ig1-2) V189R with Myc-tagged BCL10 in HEK293T cells. This experimental design represents the reverse co-IP strategy as compared to Figure 1E (anti-HA versus anti-Myc IP). Co-IPs are representative of 2-3 independent experiments.

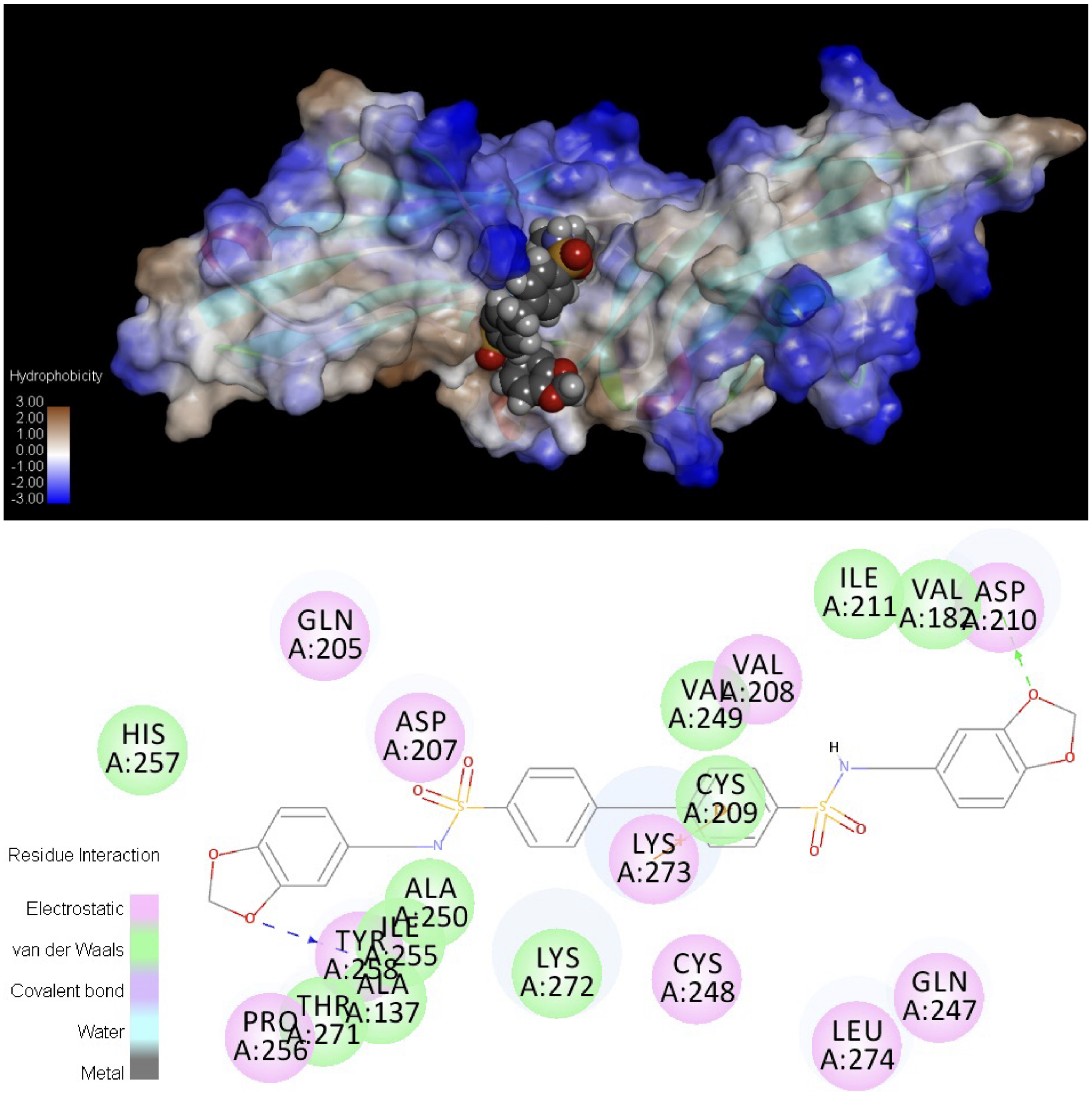

Supplemental Figure 2. Computational docking of M1i-124 to the tandem MALT1 Ig domains. Lead compound, M1i-124 is shown docked to the MALT1(Ig1-2) domains (PDB ID:3K0W), rendered to show surface hydrophobicity. The putative interactions between M1i-124 and specific MALT1 amino acids are illustrated below. These putative interactions were used to guide the site directed mutagenesis studies presented in Figure 1, which largely support the predicted interactions.

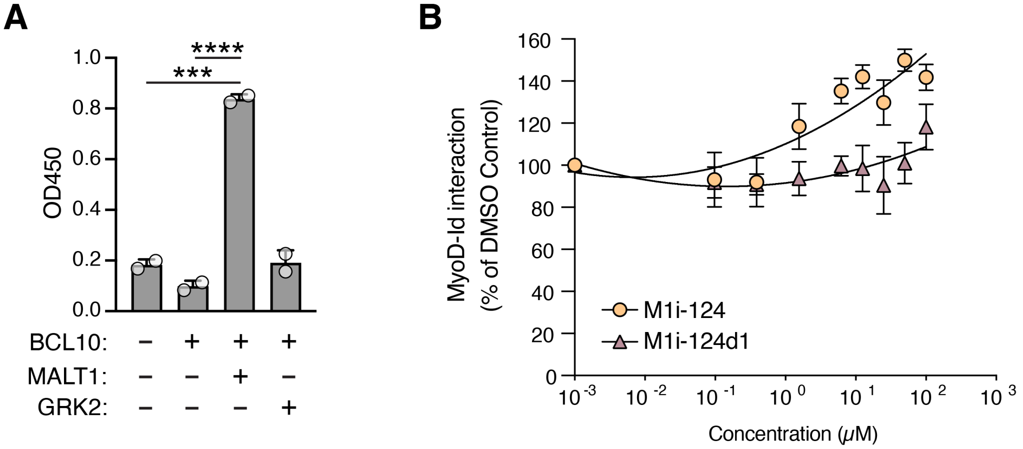

Supplemental Figure 3. M1i-124 and M1i-124d1 inhibit binding of MALT1 to BCL10 but not binding of Id to MyoD.
(A) Recombinant full-length MALT1 binds to immobilized BCL10 while GRK2 does not bind to immobilized BCL10 in the ELISA-based assay system depicted in Figure 2G (mean ± SEM; n=2). Statistical analyses were performed with one-way Anova and Dunnett's multiple comparison test. ***P<0.001, ****P<0.0001.
(B) M1i-124 and M1i-124d1 do not inhibit binding of Id to immobilized MyoD in the ELISA-based assay system (mean ± SEM; n=3).

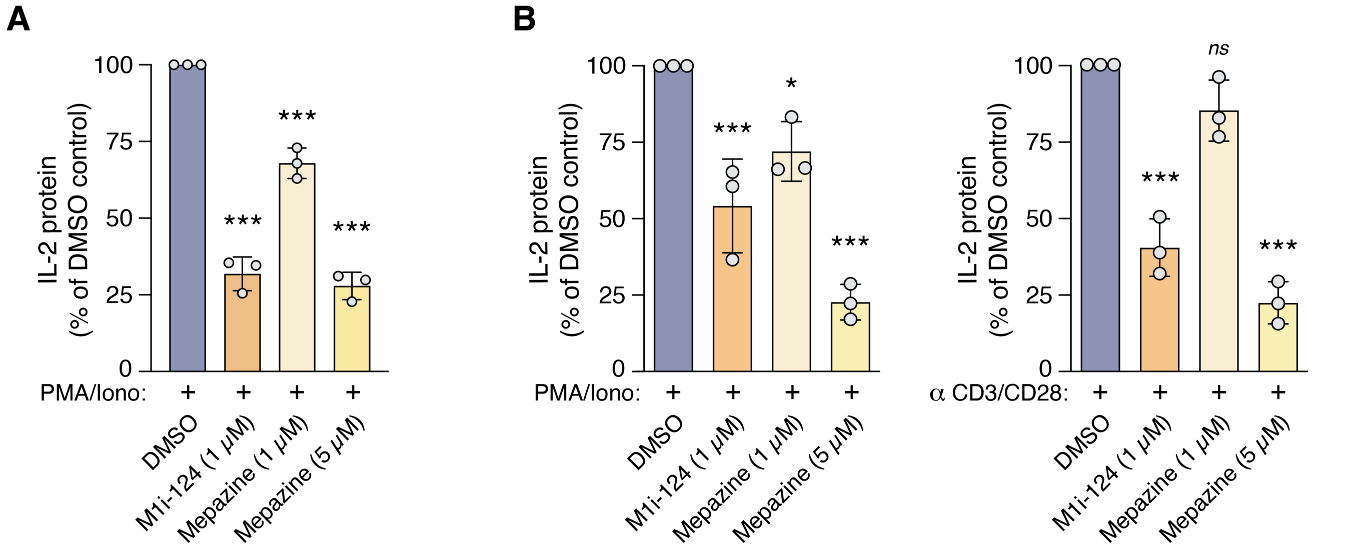

Supplemental Figure 4. M1i-124 is a potent inhibitor of IL-2 secretion from stimulated Jurkat T-cells and mouse splenocytes.
(A) ELISA data showing the effect of M1i-124 (1 µM) or Mepazine (1 µM or 5 µM) on IL-2 secretion from Jurkat T cells stimulated with PMA/Iono (mean ± SD; n=3). **(B)** ELISA data on secreted IL-2 from mouse splenocytes treated with M1i-124 (1 µM) or Mepazine (5 µM) and stimulated with PMA/Iono or anti-CD3/CD28 (mean ± SD; n=3). Statistical analyses were performed with one-way Anova and Dunnett's multiple comparison test. For all panels, *P<0.05, ***P<0.001.

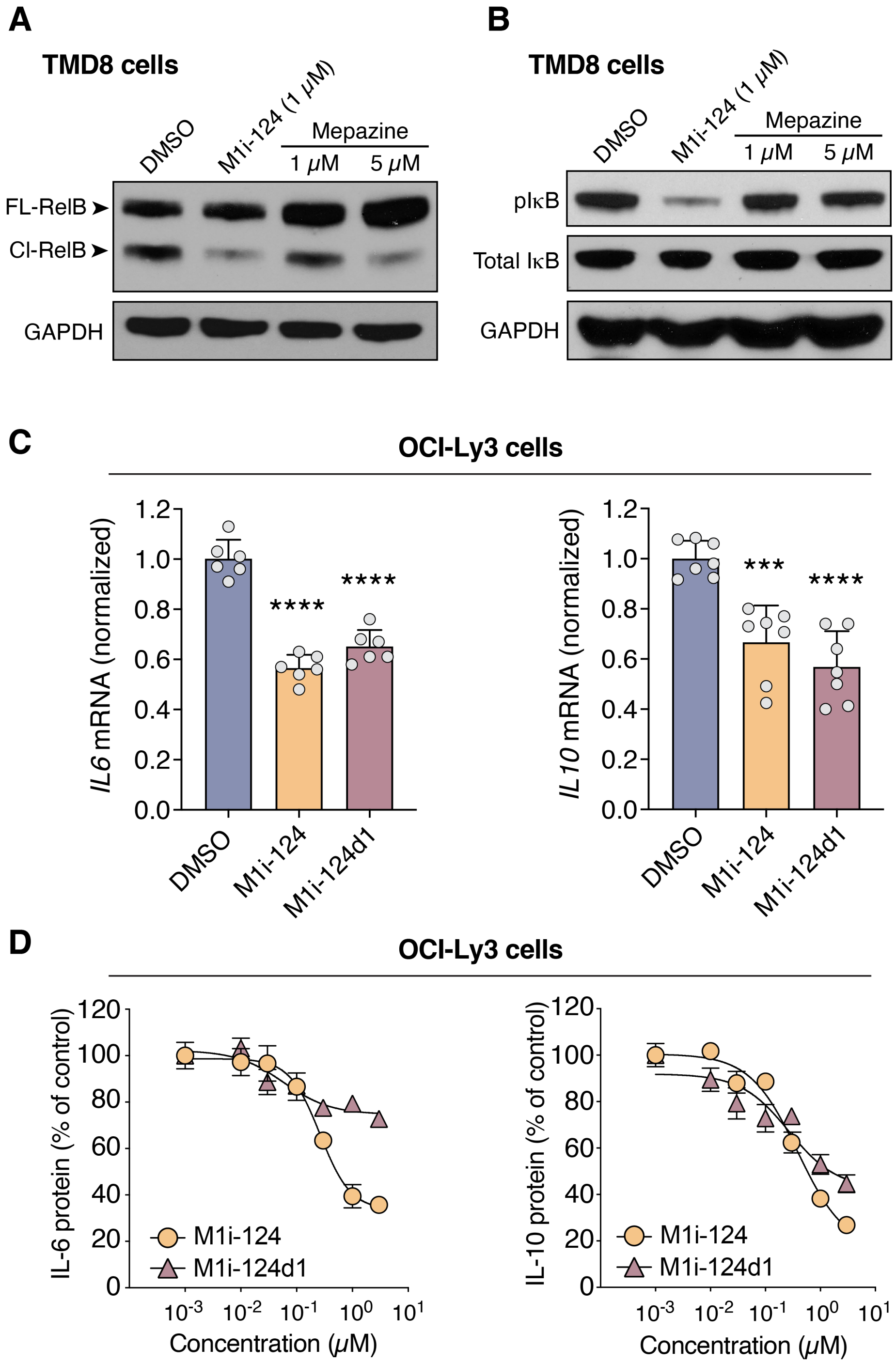

Supplemental Figure 5. M1i-124 inhibits MALT1 activity in ABC-DLBCL cells.
(A and B) The effect of M1i-124 (1 µM) versus Mepazine (1 µM or 5 µM) on constitutive MALT1-dependent protease and scaffolding activities in TMD8 cells, as measured by RelB cleavage (A) and IκB phosphorylation (B), respectively. **(C)** IL-6 and IL-10 mRNA levels in OCI-Ly3 cells treated with 1 µM M1i-124 or M1i-124d1 (mean ± SD; n=6-7) for 4 (IL-6) or 6 hours (IL-10). Statistical analyses were performed using one-way ANOVA and Dunnett's multiple comparisons test. ***P<0.001, ****P<0.0001. **(D)** Dose-dependent inhibition of IL-6 and IL-10 secretion from OCI-Ly3 cells upon treatment with M1i-124 or M1i-124d1 (mean ± SD; n=3).

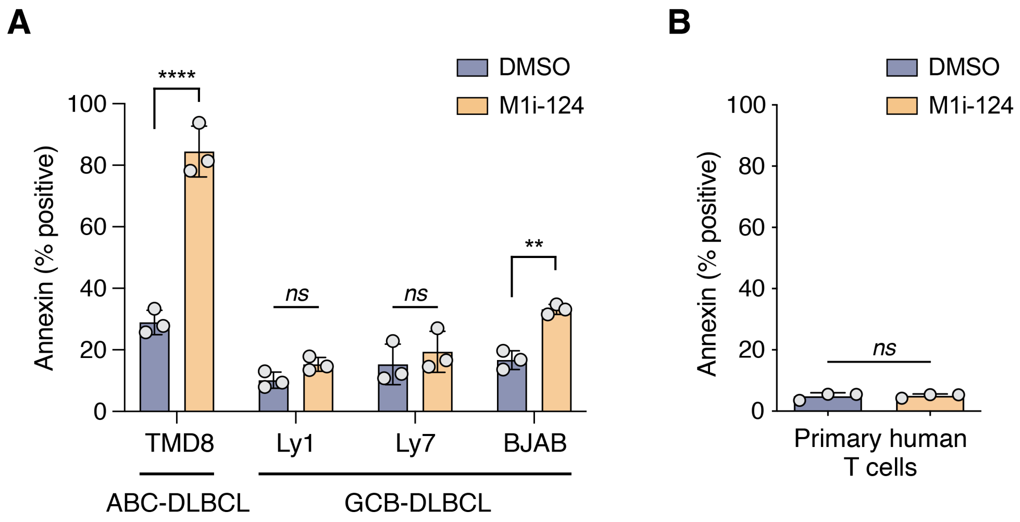

Supplemental Figure 6. M1i-124 is selective cytotoxic to ABC-DLBCL lymphoma cells.
(A) The effect of M1i-124 on apoptosis of lymphoma cell lines was assessed by annexin staining and flow-based quantification after 5 days of treatment. Cell lines evaluated included TMD8 cells (ABC-DLBCL) as well as OCI-Ly1, OCI-Ly7, and BJAB cells (GCB-DLBCL). Data represent mean ± SD; n=3. Statistical analyses were performed using two-way ANOVA with Sidak's multiple comparisons. **P<0.01, ****P<0.0001. **(B)**. Apoptosis of primary human peripheral T-cells was similarly assessed after 6 days of M1i-124 treatment. Each data point represents the analysis of T-cells from an individual donor, repeated twice. Data represent mean ± SD. Statistical analysis was performed using t-test.

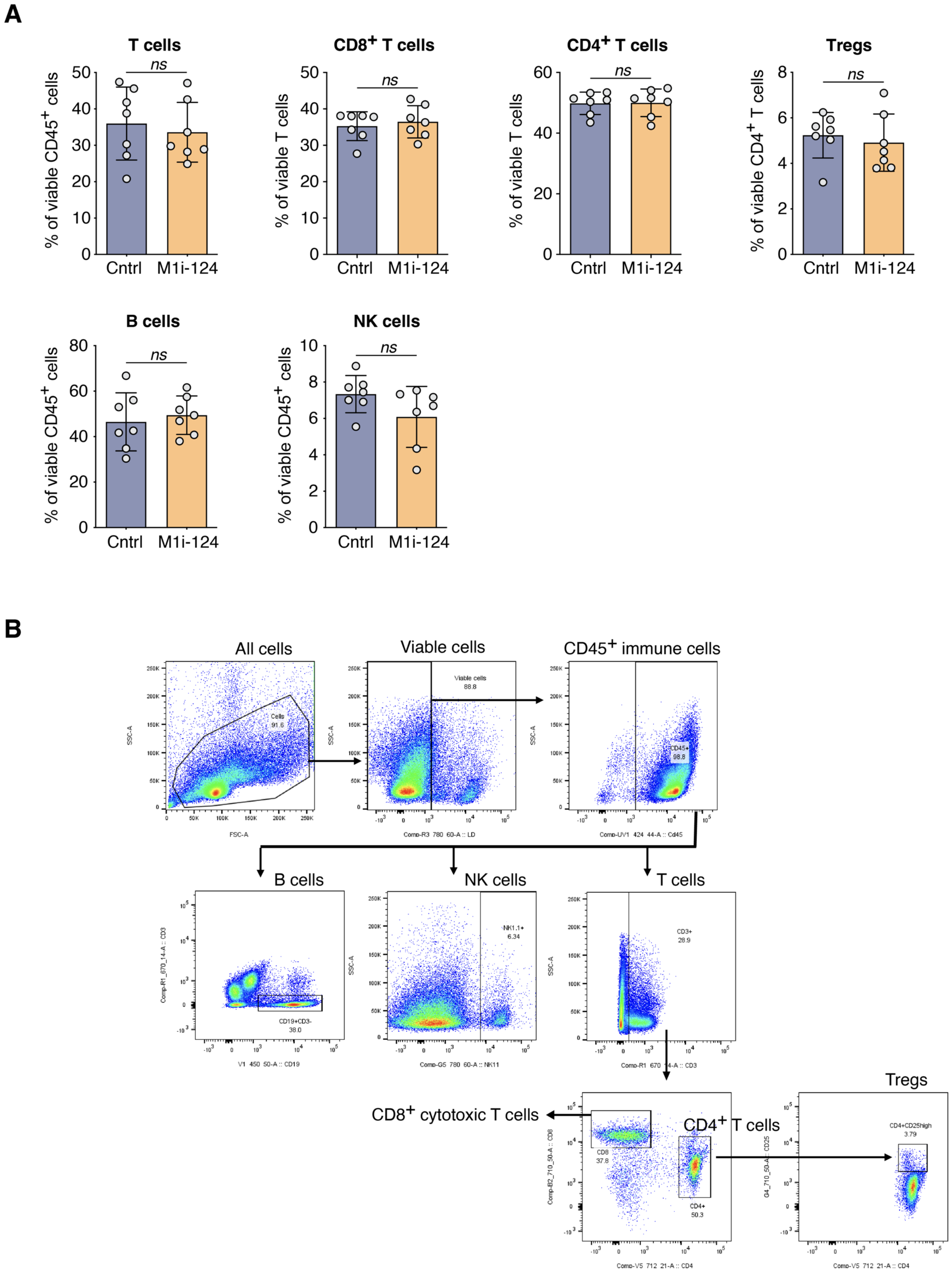

Supplemental Figure 7. Chronic M1i-124 administration does not impact the abundance of selected immune cell subsets within the spleens of treated C57/BL6 mice.
(A) Flow cytometric quantification of immune cells within spleens harvested from mice treated daily with either M1i-124 or vehicle control for a total of 12 days (mean ± SD; n=7). Statistical analyses were performed using unpaired students t-test. **(B)** Gating strategies for flow cytometric analyses presented in panel (A).

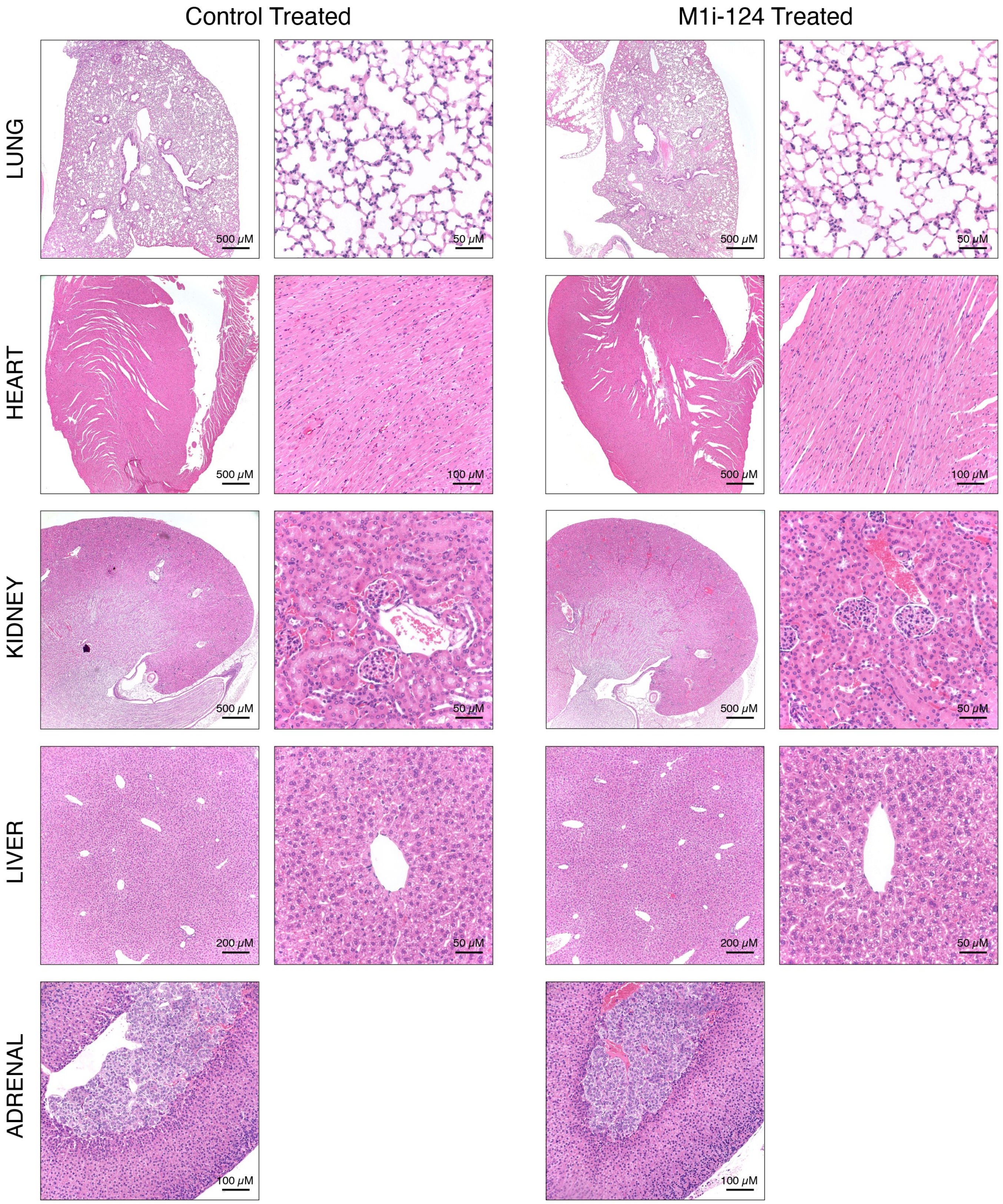

Supplemental Figure 8: M1i-124 does not cause histologic evidence of organ toxicity in mice. H&E sections of lung, heart, kidney, liver, and adrenal gland from NOD/SCID mice in the TMD8 mouse xenograft experiment. Mice received 12 daily IP injections of DMSO control or M1i-124. Organs were obtained at the time of tumor harvest, approximately 24 hours after the last injection of DMSO or M1i-124.

Cell line	Compound	IC50 (μM)	
		IL-6 inhibition	IL-10 inhibition
TMD8	M1i-124	1.3	0.5
	M1i-124d1	0.8	0.2
OCI-Ly3	M1i-124	0.8	0.7
	M1i-124d1	8.8	2.2

Supplemental Table 1. Activity of M1i-124 and M1i-124d1 in blocking cytokine secretion from ABC-DLBCL cells. IC50 values for inhibition of IL-6 and IL-10 secretion from TMD8 and OCI-Ly3 cell lines. Results for both M1i-124 and M1i-124d1 are included.

Target Class	Assay Target	Mode	Result Type	(µM)
GPCR	ADORA2A	Agonist	EC50	>10
GPCR	ADRA1A	Agonist	EC50	>10
GPCR	AVPR1A	Agonist	EC50	>10
GPCR	CCKAR	Agonist	EC50	>10
GPCR	CHRM1	Agonist	EC50	>10
GPCR	CHRM3	Agonist	EC50	>10
GPCR	EDNRA	Agonist	EC50	>10
GPCR	HRH1	Agonist	EC50	>10
GPCR	HTR2A	Agonist	EC50	>10
GPCR	HTR2B	Agonist	EC50	>10
GPCR	ADORA2A	Antagonist	IC50	>10
GPCR	ADRA1A	Antagonist	IC50	>10
GPCR	AVPR1A	Antagonist	IC50	>10
GPCR	CCKAR	Antagonist	IC50	>10
GPCR	CHRM1	Antagonist	IC50	>10
GPCR	CHRM3	Antagonist	IC50	>10
GPCR	EDNRA	Antagonist	IC50	>10
GPCR	HRH1	Antagonist	IC50	>10
GPCR	HTR2A	Antagonist	IC50	>10
GPCR	HTR2B	Antagonist	IC50	>10
GPCR	ADRA2A	Agonist	EC50	>10
GPCR	ADRB1	Agonist	EC50	>10
GPCR	ADRB2	Agonist	EC50	>10
GPCR	CHRM2	Agonist	EC50	>10
GPCR	CNR1	Agonist	EC50	>10
GPCR	CNR2	Agonist	EC50	>10
GPCR	DRD1	Agonist	EC50	>10
GPCR	DRD2S	Agonist	EC50	>10
GPCR	HRH2	Agonist	EC50	>10
GPCR	HTR1A	Agonist	EC50	>10
GPCR	HTR1B	Agonist	EC50	>10
GPCR	OPRD1	Agonist	EC50	>10
GPCR	OPRK1	Agonist	EC50	>10
GPCR	OPRM1	Agonist	EC50	>10
GPCR	ADRA2A	Antagonist	IC50	>10
GPCR	ADRB1	Antagonist	IC50	0.8
GPCR	ADRB2	Antagonist	IC50	0.6
GPCR	CHRM2	Antagonist	IC50	1.2
GPCR	CNR1	Antagonist	IC50	0.0
GPCR	CNR2	Antagonist	IC50	9.9
GPCR	DRD1	Antagonist	IC50	1.9
GPCR	DRD2S	Antagonist	IC50	>10
GPCR	HRH2	Antagonist	IC50	2.3
GPCR	HTR1A	Antagonist	IC50	>10
GPCR	HTR1B	Antagonist	IC50	>10
GPCR	OPRD1	Antagonist	IC50	>10
GPCR	OPRK1	Antagonist	IC50	>10
GPCR	OPRM1	Antagonist	IC50	3.7

Target Class	Assay Target	Mode	Result Type	(µM)
Ion Channel	CAV1.2	Blocker	IC50	>10
Ion Channel	GABAA	Blocker	IC50	>10
Ion Channel	hERG	Blocker	IC50	>10
Ion Channel	HTR3A	Blocker	IC50	>10
Ion Channel	KvLQT1/minK	Blocker	IC50	1.8
Ion Channel	nAChR(a4/b2)	Blocker	IC50	4.0
Ion Channel	NAV1.5	Blocker	IC50	>10
Ion Channel	NMDAR (1A/2B)	Blocker	IC50	>10
Ion Channel	GABAA	Opener	EC50	>10
Ion Channel	HTR3A	Opener	EC50	>10
Ion Channel	KvLQT1/minK	Opener	EC50	>10
Ion Channel	nAChR(a4/b2)	Opener	EC50	>10
Ion Channel	NMDAR (1A/2B)	Opener	EC50	>10
Kinases	INSR	Inhibitor	IC50	>10
Kinases	LCK	Inhibitor	IC50	>10
Kinases	ROCK1	Inhibitor	IC50	>10
Kinases	VEGFR2	Inhibitor	IC50	>10
NHR	AR	Agonist	EC50	>10
NHR	AR	Antagonist	IC50	>10
NHR	GR	Agonist	EC50	>10
NHR	GR	Antagonist	IC50	>10
Non-Kinase Enzymes	AChE	Inhibitor	IC50	>10
Non-Kinase Enzymes	COX1	Inhibitor	IC50	>10
Non-Kinase Enzymes	COX2	Inhibitor	IC50	>10
Non-Kinase Enzymes	MAOA	Inhibitor	IC50	>10
Non-Kinase Enzymes	PDE3A	Inhibitor	IC50	>10
Non-Kinase Enzymes	PDE4D2	Inhibitor	IC50	>10
Transporter	DAT	Blocker	IC50	>10
Transporter	NET	Blocker	IC50	>10
Transporter	SERT	Blocker	IC50	>10

Supplemental Table 2. In vitro assessment of off-target activity shows an overall safe pharmacological profile for M1i-124. EC50 or IC50 for activity against select GPCRs, ion channels, nuclear hormone receptors, kinases, and neurotransmitter transporters in an off-target activity screen of M1i-124 (Eurofins SAFETYscan78). When unmeasurable, the EC50 or IC50 for an indicated target is listed as >10 µM.
