# Supplementary material for: A small-molecule inhibitor of BCL10-MALT1 interaction abrogates progression of diffuse large B cell lymphoma
Source: J Clin Invest. 2025 Apr 15;135(8):e164573. doi: 10.1172/JCI164573 (PMC11996864; doi:10.1172/JCI164573)
Supplement: Unedited blot and gel images [file jci-135-164573-s324.pdf]

Full unedited gels for Figure 1

B

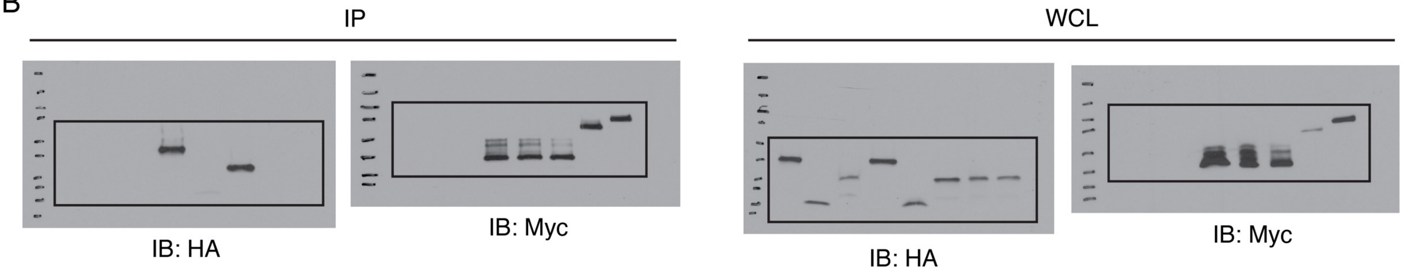


C

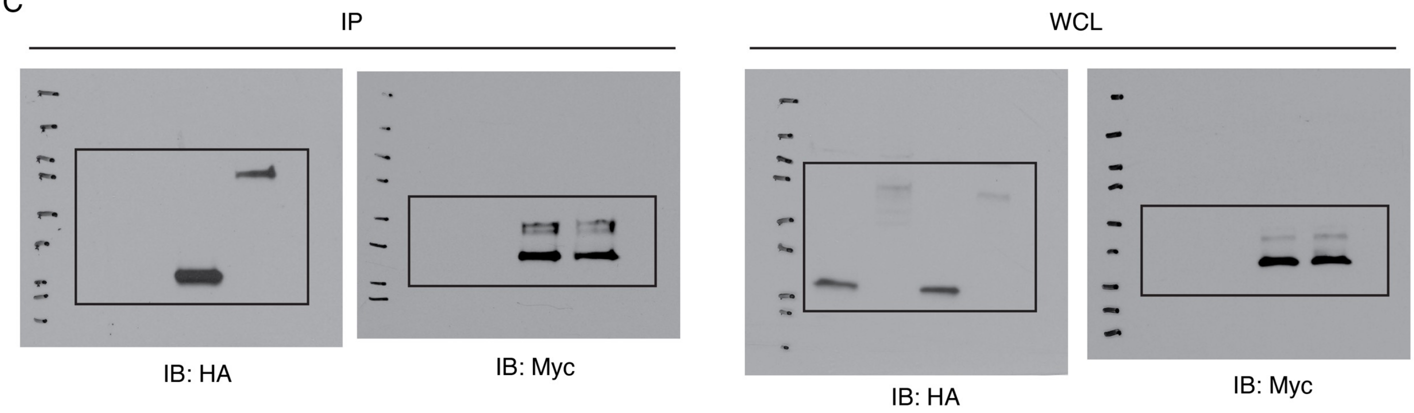


E

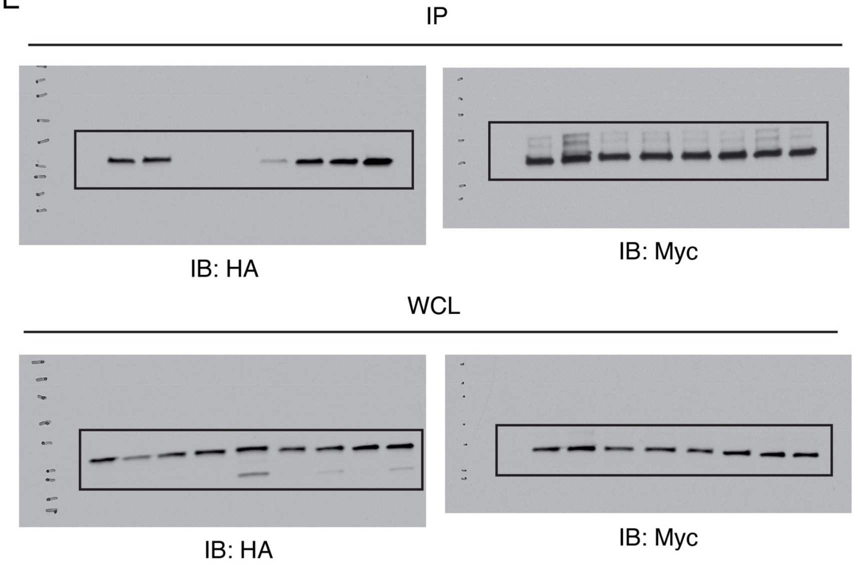


F

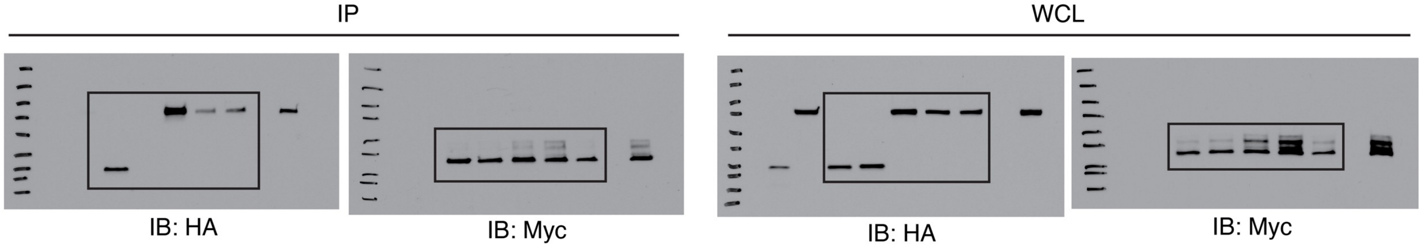

Full unedited gels for Figure 3

B

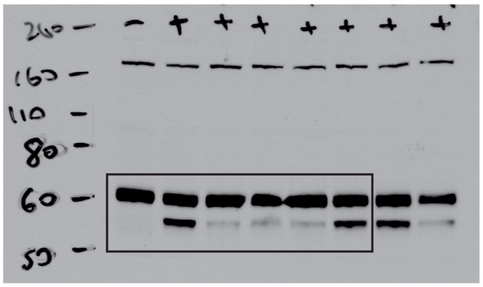

IB: RelB

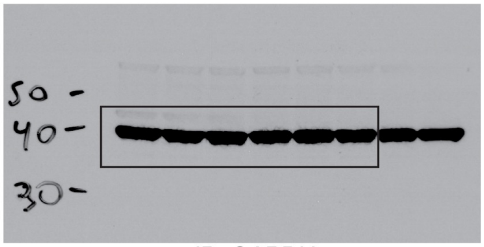

IB: GAPDH

C

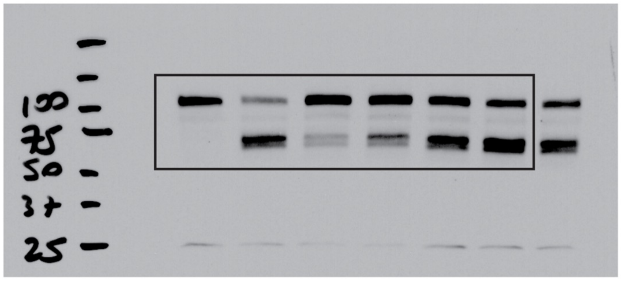

IB: N4BP1

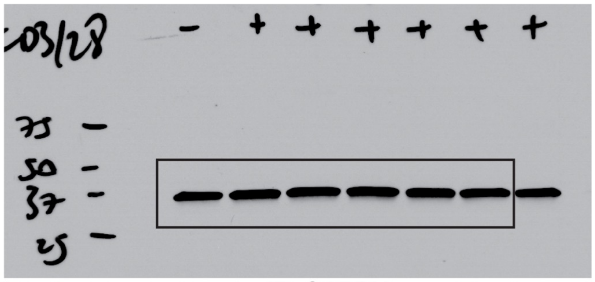

IB: GAPDH

D

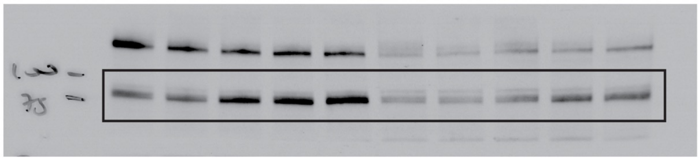

IB: pIKKα/β

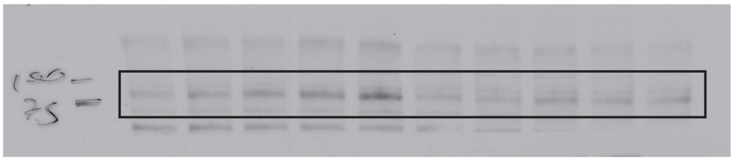

IB: pIKKα/β

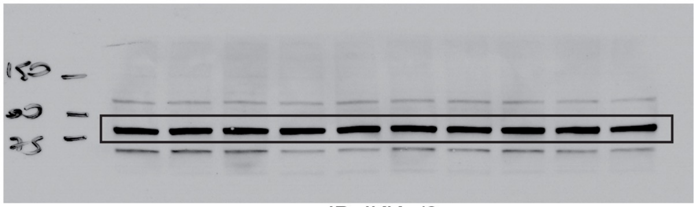

IB: IKKα/β

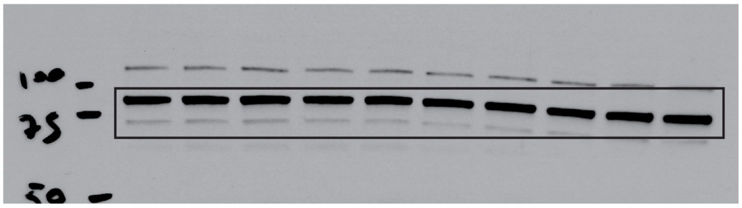

IB: IKKα/β

E

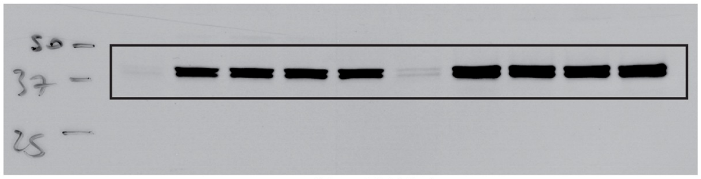

IB: pERK1/2

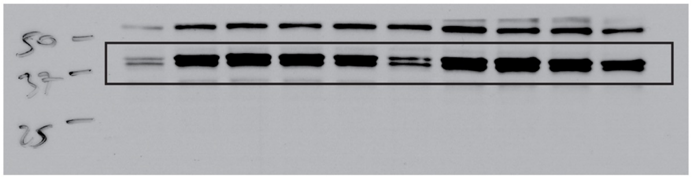

IB: pERK1/2

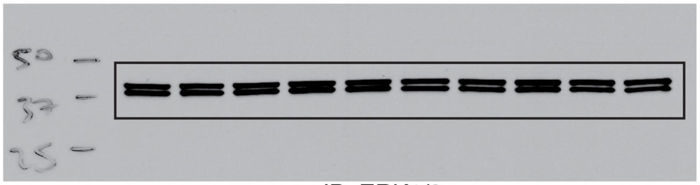

IB: ERK1/2

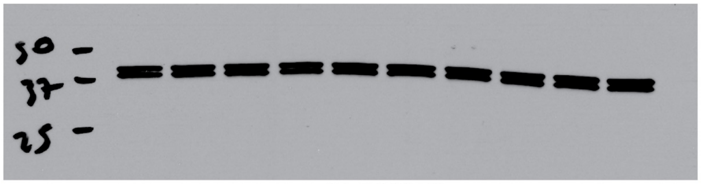

IB: ERK1/2

Full unedited gels for Figure 4

A

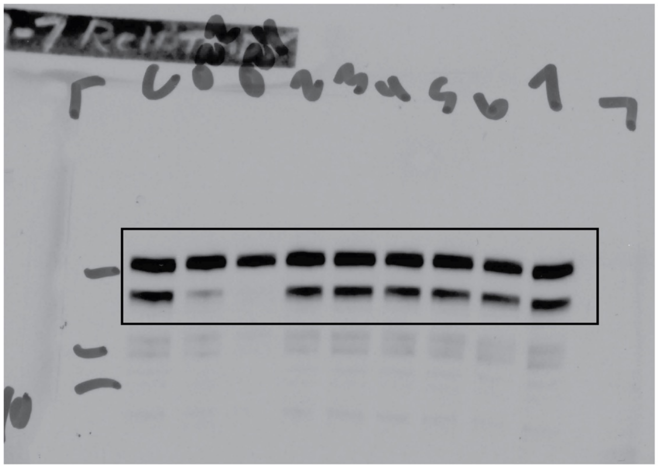

IB: RelB

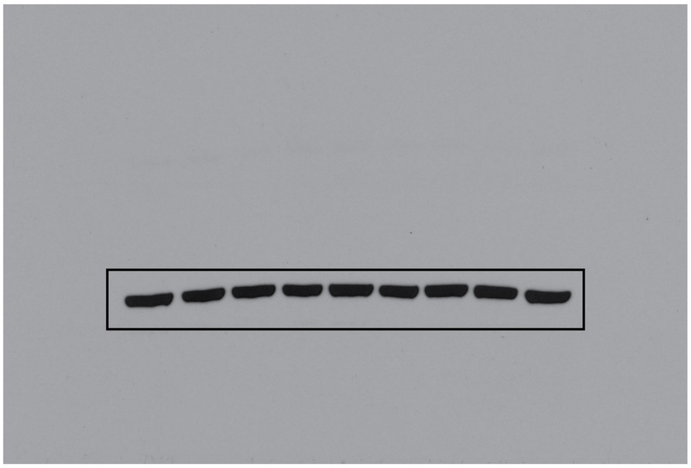

IB: GAPDH

B

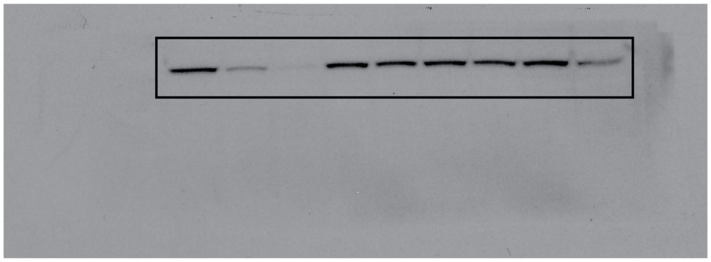

IB: pIκB

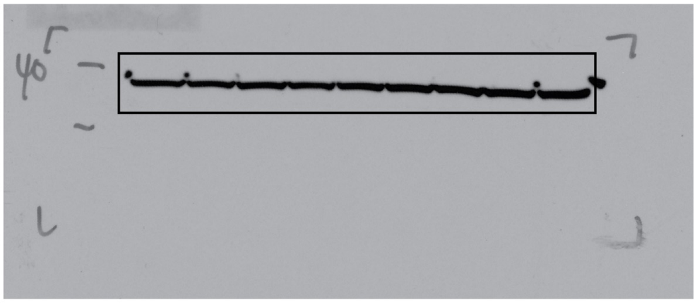

IB: GAPDH

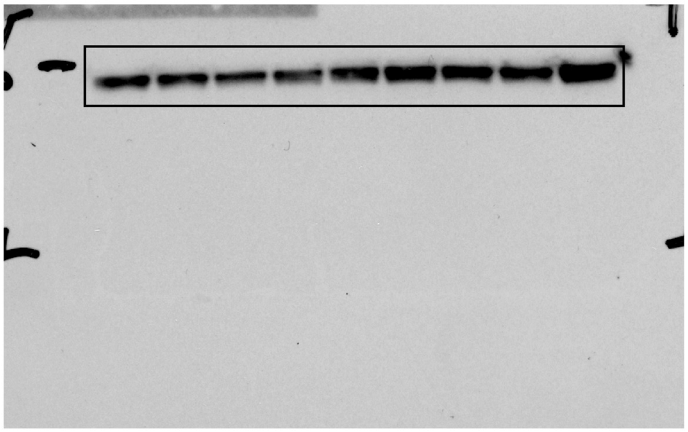

IB: Total IκB

Full unedited gels for Figure 5

A

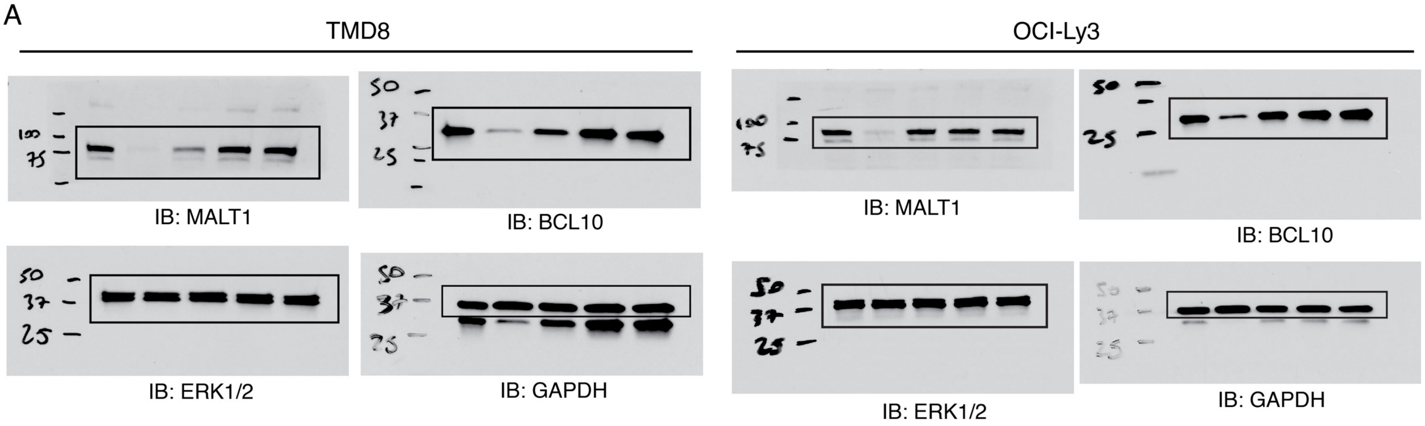


B

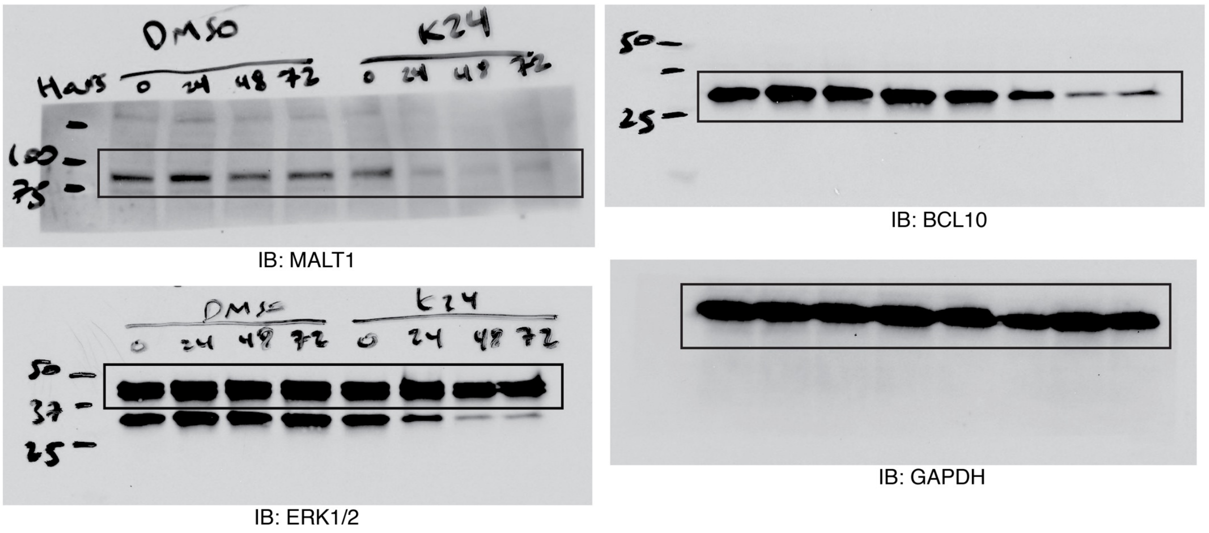


C

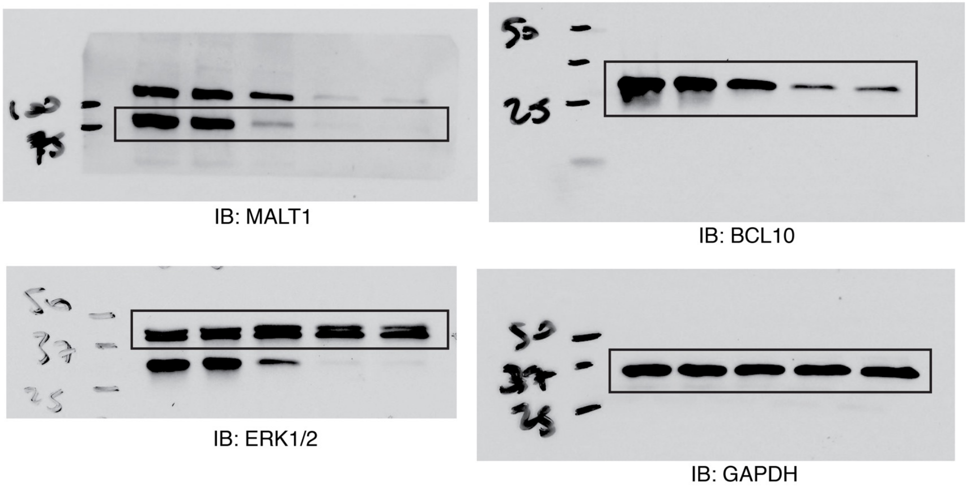

Full unedited gels for Supplemental Figure 1

C

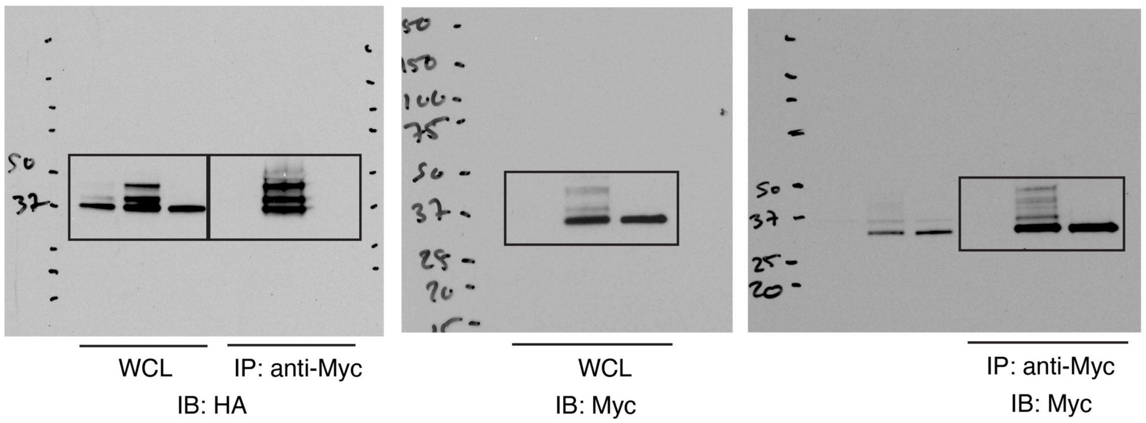


D

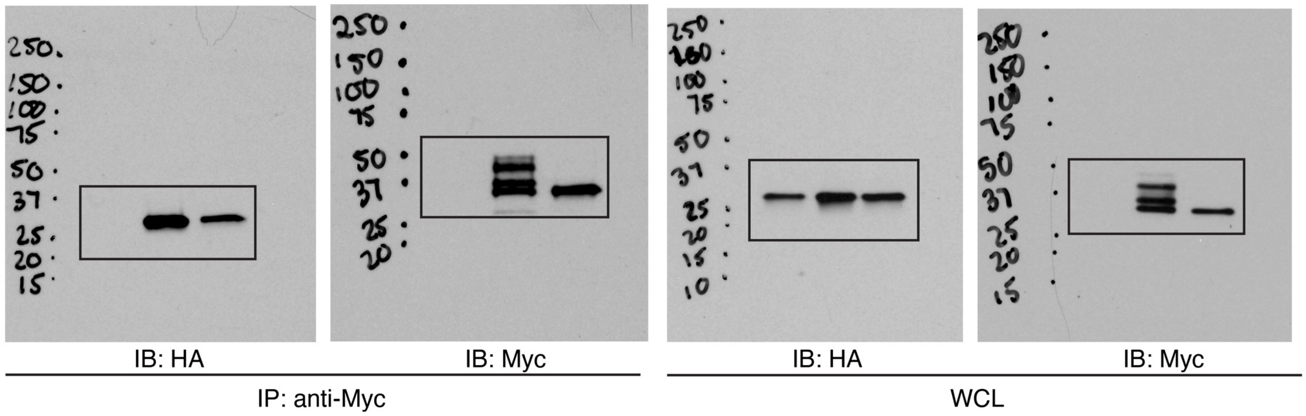


E

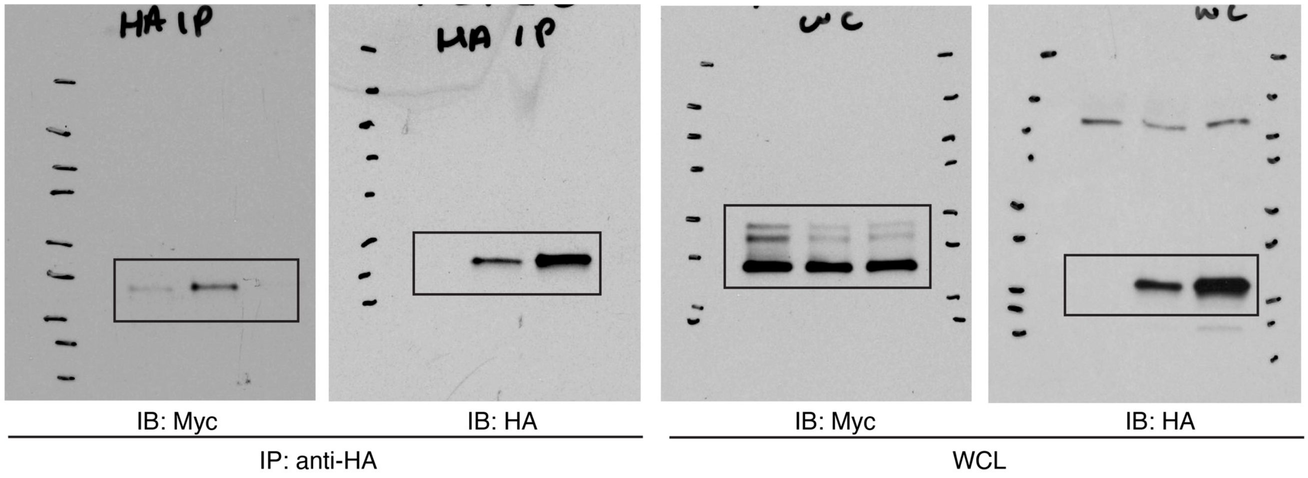

Full unedited gels for Supplemental Figure 5

A

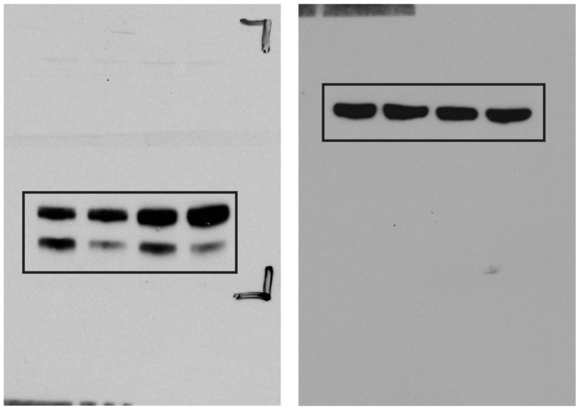

IB: RelB IB: GAPDH

B

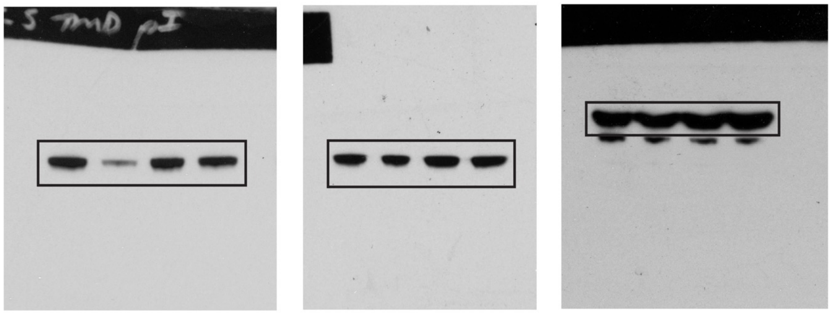

IB: pIκB IB: Total IκB IB: GAPDH
